# Cerebrospinal Fluid Biomarkers in Cerebral Amyloid Angiopathy

**DOI:** 10.3233/JAD-191254

**Published:** 2020-04-21

**Authors:** Gargi Banerjee, Gareth Ambler, Ashvini Keshavan, Ross W. Paterson, Martha S. Foiani, Jamie Toombs, Amanda Heslegrave, John C. Dickson, Francesco Fraioli, Ashley M. Groves, Michael P. Lunn, Nick C. Fox, Henrik Zetterberg, Jonathan M. Schott, David J. Werring

**Affiliations:** a Stroke Research Centre, Department of Brain Repair and Rehabilitation, UCL Queen Square Institute of Neurology and the National Hospital for Neurology and Neurosurgery, London, UK; b Department of Statistical Science, University College London, Gower Street, London, UK; c Dementia Research Centre, Department of Neurodegenerative Disease, UCL Queen Square Institute of Neurology, London, UK; d Department of Neurodegenerative Disease, UCL Queen Square Institute of Neurology, London, UK; e UK Dementia Research Institute at UCL, London, UK; f Institute of Nuclear Medicine, UCL and University College Hospital, London, UK; g MRC Centre for Neuromuscular Disease, National Hospital for Neurology and Neurosurgery, London, UK; h Clinical Neurochemistry Laboratory, Sahlgrenska University Hospital, Mölndal, Sweden; i Department of Psychiatry and Neurochemistry, Institute of Neuroscience and Physiology, The Salhgrenska Academy at the University of Gothenburg, Gothenburg, Sweden

**Keywords:** Alzheimer’s disease, amyloid-β, biomarkers, cerebral amyloid angiopathy, cerebrospinal fluid

## Abstract

**Background:**

There is limited data on cerebrospinal fluid (CSF) biomarkers in sporadic amyloid-β (Aβ) cerebral amyloid angiopathy (CAA).

**Objective:**

To determine the profile of biomarkers relevant to neurodegenerative disease in the CSF of patients with CAA.

**Methods:**

We performed a detailed comparison of CSF markers, comparing patients with CAA, Alzheimer’s disease (AD), and control (CS) participants, recruited from the Biomarkers and Outcomes in CAA (BOCAA) study, and a Specialist Cognitive Disorders Service.

**Results:**

We included 10 CAA, 20 AD, and 10 CS participants (mean age 68.6, 62.5, and 62.2 years, respectively). In unadjusted analyses, CAA patients had a distinctive CSF biomarker profile, with significantly lower (*p* < 0.01) median concentrations of Aβ_38_, Aβ_40_, Aβ_42_, sAβPP*α*, and sAβPPβ. CAA patients had higher levels of neurofilament light (NFL) than the CS group (*p* < 0.01), but there were no significant differences in CSF total tau, phospho-tau, soluble TREM2 (sTREM2), or neurogranin concentrations. AD patients had higher total tau, phospho-tau and neurogranin than CS and CAA groups. In age-adjusted analyses, differences for the CAA group remained for Aβ_38_, Aβ_40_, Aβ_42_, and sAβPPβ. Comparing CAA patients with amyloid-PET positive (*n* = 5) and negative (*n* = 5) scans, PET positive individuals had lower (*p* < 0.05) concentrations of CSF Aβ_42_, and higher total tau, phospho-tau, NFL, and neurogranin concentrations, consistent with an “AD-like” profile.

**Conclusion:**

CAA has a characteristic biomarker profile, suggestive of a global, rather than selective, accumulation of amyloid species; we also provide evidence of different phenotypes according to amyloid-PET positivity. Further replication and validation of these preliminary findings in larger cohorts is needed.

## INTRODUCTION

Sporadic amyloid-β (Aβ) cerebral amyloid angiopathy (CAA) can be reliably diagnosed during life using the clinico-radiological Boston criteria [1, 2], but nearly all of its associated imaging features are likely to be irreversible markers of late stage disease [3]. Body fluid biomarkers, using either cerebrospinal fluid (CSF) or blood, are of interest as important candidate biomarkers which can be sampled repeatedly, allow for measurement of a variety of different disease-related processes, and provide insights into disease dynamics.

To date, most data on CSF biomarkers in CAA have focused on Aβ (Aβ_40_, Aβ_42_) and tau (total tau, t-tau, and phospho-tau, p-tau) measures. However, there is limited information on other amyloid species in CAA, including smaller proteins such as Aβ_38_, soluble amyloid-β protein precursor (sAβPP) *α* and β. Additionally, a number of newer fluid biomarkers are of potential interest in CAA. Neurofilament light (NFL), soluble TREM2 (sTREM2) and neurogranin are promising new biomarkers for Alzheimer’s disease (AD), but it is not clear whether they are specific for parenchymal amyloid.

Our aim was to perform a detailed comparison of amyloid markers (Aβ_38_, Aβ_40_, Aβ_42_, sAβPP*α*, and sAβPPβ) and other markers studied in neurodegenerative disease (t-tau, p-tau, NFL, sTREM2, and neurogranin) in the CSF of patients with AD, CAA, and control (CS) participants in an exploratory hypothesis-generating study. To explore the heterogeneity of CAA CSF profile and relationship with parenchymal amyloid deposition, we then performed *post-hoc* analyses comparing the CSF profiles of CAA patients with amyloid-PET positive and negative scans.

## MATERIALS AND METHODS

### Patient selection

Participants were included from two sources. Firstly, we included participants from the cross-sectional prospective observational BOCAA (Biomarkers and Outcomes in Cerebral Amyloid Angiopathy) study (10 patients with CAA, 5 CS participants). Ethical approval for the BOCAA study was granted in October 2015 by the NHS Health Research Authority London (REC reference 15/LO/1443). Secondly, we included samples collected by the Specialist Cognitive Disorders Service at the NHNN, University College London Hospitals (UCLH) NHS Trust, London, UK (20 samples from patients with AD, 5 samples from age-matched CS participants). This study was approved by the Regional Ethics Committee at UCL.

In all cases, informed written consent was obtained for each participant, and inclusion criteria were standardized to be consistent with the BOCAA study (further details below).

#### Patients with CAA

All patients with CAA were recruited from the BOCAA study [4]. Consecutive patients with CAA were identified from a prospectively collected research database. Patients with CAA all met at least probable modified Boston Criteria [2], and were not included if they had evidence of co-existing AD or deep perforator (hypertensive) arteriopathy [4]. Further inclusion criteria were: age ≥55 years, Mini-Mental State Examination (MMSE) score ≥23, modified Rankin scale (mRS) ≤3 and capacity to give informed consent. Those with contraindications to PET or MRI scanning or lumbar puncture were excluded.

#### Control (CS) participants

CS participants were included from two sources. In all cases, CS participants were required to have no prior history of significant neurological disease. Further inclusion criteria were: age ≥55 years, MMSE score ≥23, and modified Rankin scale (mRS) ≤3.

Firstly, we included CS participants recruited as part of the BOCAA study, where patient partners were invited to participate as healthy volunteers. CS participants were also identified from a prospectively collected database of patients attending the ambulatory transient ischemic attack (TIA) service, provided by the NHNN; patients whose final diagnosis was not stroke, TIA, or any other significant neurological condition (and met the inclusion and exclusion criteria) were invited to participate. Those with contraindications to PET or MRI scanning or lumbar puncture were excluded.

Secondly, we included samples collected by the Specialist Cognitive Disorders Service at the National Hospital of Neurology and Neurosurgery (NHNN). Samples for age-matched CS participants were included if their final diagnosis, made on the basis of clinical assessment, imaging, and CSF, was not one of dementia or any other neurodegenerative condition [5]. MR imaging (acquired as part of routine clinical care) was reviewed for evidence of previous infarction (including lacunes), cerebral microbleeds, and cortical superficial siderosis; participant samples were only included in the absence of all of these features. Atrophy (medial temporal [6] and global cortical [7]) and white matter hyperintensities [8] were assessed on brain imaging, and those with evidence of moderate or severe grades of these imaging features were excluded.

#### Patients with Alzheimer’s disease

Patients with AD presented with “typical” [9] amnestic symptoms, were aged ≥55 years, and had a final diagnosis (on the basis of clinical assessment, imaging, and CSF) that was in keeping with AD; additionally, all imaging was reviewed for the presence of cerebral microbleeds and cortical superficial siderosis, and samples from patients with these features were not included (in order to avoid patients with mixed CAA and AD pathology). The CSF criteria for AD diagnosis were the presence of a t-tau/Aβ_42_ ratio >0.88 together with Aβ_42_ < 630 pg/ml [5].

### CSF analysis

All CSF analyses were performed by the Biomarker Laboratory of the UK Dementia Research Institute at UCL (Group Lead: Professor Henrik Zetterberg). CSF was collected, processed, and stored according to standardized procedures, and was identical for all diagnostic groups [10]. Samples were collected in polypropylene tubes, immediately transported to the laboratory by hand, and centrifuged (at 1,750 for 5 min at 4°C) within 30 min from collection; samples were then aliquoted and stored at –80°C until testing. All biochemical assays were performed by operators blinded to the clinical diagnosis.

#### Amyloid markers

Aβ_38_, Aβ_40_, and Aβ_42_ were measured by electrochemiluminescence (ECL) using a Meso Scale Discovery V-PLEX Aβ peptide panel 1(6E10) kit, according to the manufacturer’s instructions (MSD, Rockville, MD). Briefly, samples were diluted 1 : 2 with diluent 35 and added in duplicate to microplate wells coated with mouse monoclonal peptide specific capture antibodies for human Aβx-38/x-40/x-42. Samples were incubated with anti-Aβ (amino acids 1–16 epitope) antibody (6E10 clone) as the detection antibody conjugated with an electrically excitable SULFO-TAG. Concentrations were calculated from ECL signal using a four-parameter logistic curve fitting method with the MSD Workbench software package. Intra-assay CVs (coefficients of variation) were less than 10%. All samples were measured on the same day by a single operator using the same reagents.

sAβPP*α* and sAβPPβ were measured by ECL using a Meso Scale Discovery sAβPP*α*/sAβPPβ Kit, according to the manufacturer’s instructions (MSD, Rockville, MD). Briefly, samples were diluted 1 : 4 with 1% Blocker A and added in duplicate to microplate wells coated with mouse (sAβPP*α*) and rabbit (sAβPPβ) monoclonal peptide specific capture antibodies. Samples were incubated with anti-sAβPP*α* and anti-sAβPPβ detection antibodies conjugated with an electrically excitable SULFO-TAG. Concentrations were calculated from ECL signal using a four-parameter logistic curve fitting method with the MSD Workbench software package. Intra-assay CVs were less than 20%. All samples were measured on the same day by a single operator using the same reagents.

#### Tau markers (t-tau and p-tau)

The levels of CSF t-tau and p-tau_ (181P) _ were determined using a sandwich ELISA (INNOtest^®^ hTAU-Ag p-Tau_ (181P) _; Fujirebio Europe N.V., Gent, Belgium) constructed to measure both normal tau and phosphorylated tau. Briefly, for the hTAU Ag assay, tau protein is captured from CSF samples by a monoclonal anti-tau antibody (AT120) bound to a microtiter plate. Captured tau is detected with two biotinylated tau-specific monoclonal antibodies (HT7 and BT2). Similarly, for the t-tau assay, p-tau_ (181P) _ is captured from CSF samples by anti-tau antibody HT7 bound onto a microtiter plate. Captured p-tau_ (181P) _ is detected with a biotinylated monoclonal anti-p-tau antibody (AT270). In both assays, peroxidase-labelled streptavidin and tetramethylbenzidine (TMB) substrate are also added. Peroxidase-catalyzed hydrolysis produces a colorimetric signal. Sample concentrations are extrapolated from a standard curve, fitted using a 4-parameter logistic algorithm. Intra-assay CVs were less than 20%.

#### Neurofilament light

NFL was measured using the commercially available NF-Light ELISA, according to the manufacturer’s instructions (UmanDiagnostics, Umeå, Sweden). Briefly, samples were diluted 1 : 2 with sample diluent and added in duplicate to microplate wells coated with a monoclonal capture antibody specific for NFL. Samples were incubated with a biotinylated NFL-specific monoclonal detection antibody. The detection complex was completed with the addition of horseradish peroxidase-labelled streptavidin and TMB substrate. Peroxidase-catalyzed hydrolysis produces a colorimetric signal. Sample concentrations were extrapolated from a standard curve, fitted using a 4-parameter logistic algorithm. Intra-assay CVs were less than 10%. Samples were run on two different days by different operators; the inter-assay CV was below 16%.

#### sTREM2

Samples were analyzed using an immunoassay protocol adapted from a previously published protocol [11]. Streptavidin-coated 96-well plates (Meso-Scale Discovery (MSD), Rockville, MD, USA) were blocked overnight at 4°C in block buffer (0.5% bovine serum albumin (BSA) and 0.05% Tween 20 in PBS; pH 7.4). The plates were then incubated with the biotinylated polyclonal goat anti-human TREM2 capture antibody (0.25*μ*g/ml; BAF1828, R&D Systems, Minneapolis, MN, USA) diluted in block buffer, shaking for 1 h at room temperature. They were subsequently washed five times with wash buffer (0.05% Tween 20 in PBS) and incubated for 2 h shaking at room temperature with 50*μ*L per well of either the standard curve constructed from recombinant human TREM2 protein (11084-H08H-50, Sino Biological Inc., Beijing, China) diluted in assay buffer (0.25% BSA and 0.05% Tween 20 in PBS; pH 7.4) to produce concentrations ranging between 4000 pg/ml and 62.5pg/ml, or CSF samples diluted 1 in 4 in assay buffer. Standards and CSF samples were assayed in duplicate. Plates were again washed five times with wash buffer before incubation for 1 h shaking at room temperature with the detection antibody, monoclonal mouse anti-human TREM2 antibody (1*μ*g/ml; (B-3): sc373828, Santa Cruz Biotechnology, TX, USA), diluted in block buffer. After five additional washing steps, plates were incubated with the secondary antibody (SULFO-TAG-labelled goat anti-mouse secondary antibody, R32AC-5, MSD) and incubated shaking for 1 h in the dark. Lastly, plates were washed three times with wash buffer then twice in PBS alone. The electrochemical signal was developed by adding MSD Read buffer T 4× (R92TC-2, MSD) diluted 1 in 2, and the light emission measured using the MSD Sector Imager 6000. The concentration of sTREM2 was calculated using a five-parameter logistic curve fitting method with the MSD Workbench software package. Intra-assay CVs were less than 10%, and all samples were measured on the same day by a single operator using the same reagents.

#### Neurogranin

Neurogranin was measured with the EUROIMMUN Elisa (EQ6551-9601-L) according to manufacturer’s instructions (EUROIMMUN, Lübeck, Germany). Briefly, samples were incubated with biotinylated monoclonal anti-neurogranin antibody, followed by addition to microplate wells coated with monoclonal antibodies specific for human neurogranin truncated at P75. Finally, streptavidin peroxidase conjugate was added to initiate the color-changing reaction. Intra-assay CVs were less than 10%, and all samples were measured on the same day by a single operator using the same reagents. Sample concentrations are extrapolated from a standard curve, fitted using a 5-parameter logistic algorithm.

### Amyloid-PET acquisition and interpretation

All participants in the BOCAA study underwent PET (using the amyloid ligand ^18^F-Florbetapir, Amyvid) and MR scanning, acquired using the same hybrid Siemens Biograph PET/MR scanner; the protocol for acquisition and initial processing has been previously described [12]. Visual reads were performed by a trained individual who was blind to all participant clinical details (including diagnosis).

### Statistics

Statistical analysis was performed using Stata (Version 15.1). Group characteristics were compared using one-way ANOVA (age), chi squared (sex), or Kruskal-Wallis (MMSE) tests. Median and interquartile range values were calculated for each biomarker, and, given the non-normal distribution of the data, comparisons between groups were made using the Kruskal-Wallis test. If a significant difference was identified (defined as p < 0.05), Dunn’s test was used for *post-hoc* comparisons, and a Bonferroni correction (resultant p value multiplied by 3) was applied.

In order to perform age-adjusted analyses, we used quantile regression (comparing group medians) and calculated predicted medians. We then performed *post-hoc* pairwise comparisons of the age-adjusted medians; statistical significance was defined as Bonferroni-corrected *p* < 0.05.

Finally, we performed exploratory *post-hoc* analyses comparing biomarkers between PET positive and negative CAA patients using Wilcoxon-Mann-Whitney tests.

## RESULTS

We included 20 patients with AD, 10 patients with CAA, and 10 CS participants in this analysis; baseline characteristics are shown in Table 1. Patients with CAA were older than the two other groups (mean age±SD: CAA 68.6±3.0 years, AD 62.5± 4.1 years, and CS 62.2±5.4 years). As expected, those in the AD group had a lower MMSE (median score 24, compared with 29 for CAA and CS groups).

**Table 1:** Comparison of characteristics and biomarkers by group. Group comparison p values were obtained using one-way ANOVA (age), chi squared tests (sex), or Kruskal-Wallis tests (remainder). *Post-hoc* comparisons were made using Dunn’s test; the presented p values are Bonferroni corrected

	CAA (*n* = 10)	AD (*n* = 20)	CS (*n* = 10)	Group comparison, p	*Post-hoc* comparisons; p		
					CAA/AD	CAA/CS	AD/CS
Age, y, mean (SD)	68.6 (3.0)	62.5 (4.1)	62.2 (5.4)	0.001	–	–	–
Sex, female, n (%)	2 (20%)	11 (55%)	5 (50%)	0.18	–	–	–
MMSE, median (IQR)	29 (28 to 30)	24 (19.5 to 26)	29 (29 to 30)	<0.001	–	–	–
***Biomarkers***
Aβ_38_, pg/ml, median (IQR)	1490 (1350 to 2450)	2740 (2360 to 3270)	2840 (2150 to 3280)	0.002	0.002	0.004	1.00
Aβ_40_, pg/ml, median (IQR)	3150 (2940 to 4140)	6470 (5760 to 7330)	6890 (5080 to 7600)	<0.001	<0.001	<0.001	1.00
Aβ_42_, pg/ml, median (IQR)	115 (91.4 to 134)	323 (264 to 376)	520 (280 to 814)	<0.001	<0.001	<0.001	0.37
sAβPP*α*, pg/ml, median (IQR)	88.6 (67.9 to 100)	115 (99.0 to 137)	117 (105 to 136)	0.008	0.007	0.013	1.00
sAβPPβ, pg/ml, median (IQR)	85.8 (66.3 to 104)	118 (102 to 142)	124 (97.5 to 144)	0.009	0.006	0.018	1.00
t-tau, pg/ml, median (IQR)	316 (247 to 440)	657 (497 to 869)	250 (206 to 266)	<0.001	<0.001	0.35	<0.001
p-tau, pg/ml, median (IQR)	62.1 (45.8 to 72.1)	92.8 (73.6 to 112)	49.5 (42.0 to 52.4)	<0.001	0.014	0.26	<0.001
NFL, pg/ml, median (IQR)	2780 (2390 to 8380)	2370 (1920 to 2730)	1470 (1150 to 1630)	<0.001	0.15	<0.001	0.005
sTREM2, pg/ml, median (IQR)	7040 (6240 to 9230)	6580 (5640 to 8120)	7960 (6130 to 9790)	0.52	–	–	–
Neurogranin, pg/ml, median (IQR)	432 (349 to 491)	565(454 to 702)	409 (309 to 431)	0.012	0.076	0.72	0.008

Of the patients with CAA, 5 (50%) presented with intracerebral hemorrhage, and the remainder presented with transient focal neurological episodes associated with convexity subarachnoid hemorrhage. All CAA patients had been clinically asymptomatic in the 6 months prior to their study visits. Amongst the CAA group, 7 patients (70%) showed evidence of cortical superficial siderosis (focal in three cases, disseminated in four cases) and 9 (90%) had lobar microbleeds (median number, 3.5). All CAA patients showed evidence of both deep and periventricular white matter hyperintensities on MRI (median Fazekas scores, 1 in deep and 2 in periventricular regions [8]).

### Individual markers

#### Univariable comparisons

**Fig.1:**
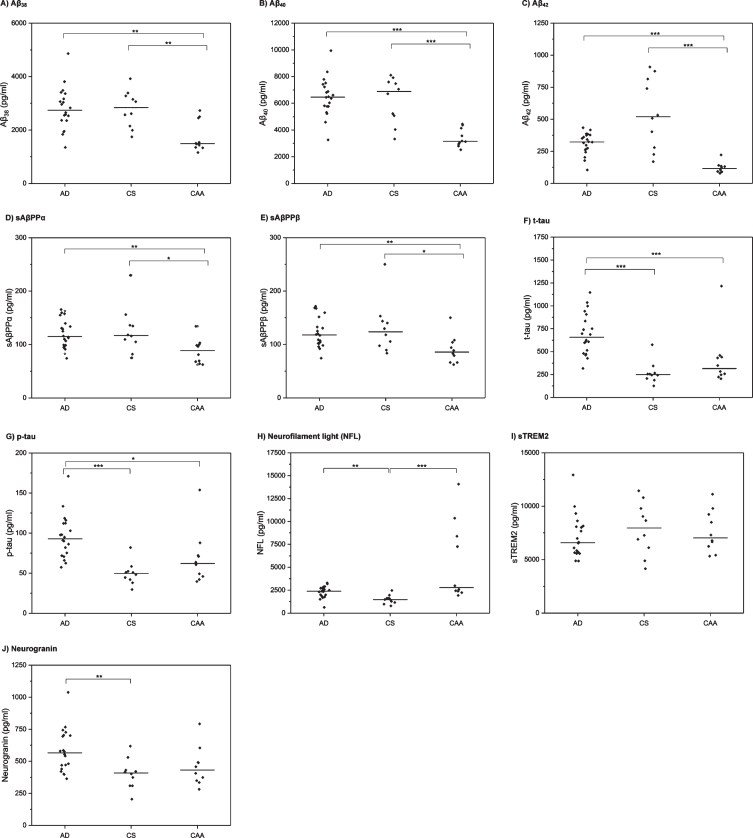
CSF biomarker profiles in AD, CAA, and control (CS) participants. Horizontal line indicates median value per group. Each diamond indicates an individual data point. *p*-values are derived from *post-hoc* Dunn’s test and have been Bonferroni-corrected. *p≤0.05, **p≤0.01, ***p≤0.001.

Univariable comparisons are shown in Table 1 and Fig. 1. There were significant differences between the three groups for the following markers: Aβ_38_, Aβ_40_, Aβ_42_, sAβPP*α*, sAβPPβ, t-tau, p-tau, NFL, and neurogranin. There was no significant difference in sTREM2 levels between the three groups.

In *post-hoc* comparisons (Table 1, Fig. 1), patients with CAA had significantly lower CSF levels of Aβ_38_, Aβ_40_, Aβ_42_, sAβPP*α* and sAβPPβ than both the AD and CS groups. Patients with AD had lower CSF Aβ_42_ than the CS group, but this was not statistically significant after Bonferroni correction; there were no significant differences between the AD and CS groups for the other amyloid markers. For the tau markers (t-tau, p-tau), there were no statistically significant differences between the CAA and CS groups; patients with AD had significantly higher levels than both CAA and CS group. Patients with both CAA and AD had significantly higher CSF NFL than the CS group; there was no statistically significant difference between the CAA and AD groups. There was no difference in neurogranin levels between CAA and either the AD or the CS groups; patients with AD had significantly higher levels of CSF neurogranin than the CS group.

#### Age-adjusted quantile regression

Results from the age-adjusted quantile regression are shown in Table 2; scatter plots demonstrating the distribution of each biomarker by age are shown in Supplementary Figure 1. For the amyloid markers, there were significant differences between the three groups for Aβ_38_, Aβ_40_, Aβ_42_, and sAβPPβ, but not sAβPP*α*. Pairwise comparisons of the age-adjusted medians found significant differences between CAA and AD groups for Aβ_38_ (higher in AD group; median difference 1,480 pg/ml), Aβ_40_ (higher in AD group; median difference 3,540 pg/ml) and sAβPPβ (higher in AD group; median difference 48.6 pg/ml); for Aβ_42_, the difference between the CAA and AD groups did not reach statistical significance (higher in AD group; median difference 162 pg/ml). There were significant differences between the CAA and CS groups for Aβ_38_ (higher in CS group; median difference 1,650 pg/ml), Aβ_40_ (median difference 4060 pg/ml), Aβ_42_ (higher in CS group; median difference 394 pg/ml), and sAβPPβ (higher in CS group; median difference 58.8 pg/ml). For Aβ_42_, there was a significant difference between the AD and CS groups (higher in CS group; median difference 232 pg/ml).

**Table 2:** Age-adjusted quantile regression (comparing medians)

Biomarker	Group	Age-adjusted difference in medians (SE)	p
Aβ_38_, pg/ml	CAA	*Reference group*	<0.001
	AD	1480 (370)
	CS	1650 (417)
Aβ_40_, pg/ml	CAA	*Reference group*	<0.001
	AD	3540 (846)
	CS	4060 (954)
Aβ_42_, pg/ml	CAA	*Reference group*	<0.001
	AD	162 (83.9)
	CS	394 (94.5)
sAβPP*α*, pg/ml	CAA	*Reference group*	0.47
	AD	18.9 (16.1)
	CS	20.0 (18.1)
sAβPPβ, pg/ml	CAA	*Reference group*	0.024
	AD	48.6 (19.1)
	CS	58.8 (21.5)
t-tau, pg/ml	CAA	*Reference group*	<0.001
	AD	357 (115)
	CS	–53.1 (129)
p-tau, pg/ml	CAA	*Reference group*	<0.001
	AD	40.9 (12.6)
	CS	–2.20 (14.2)
NFL, pg/ml	CAA	*Reference group*	0.36
	AD	–511 (844)
	CS	–1310 (952)
sTREM2, pg/ml	CAA	*Reference group*	0.55
	AD	–76.9 (1270)
	CS	1100 (1430)
Neurogranin, pg/ml	CAA	*Reference group*	0.021
	AD	210 (91.5)
	CS	24.0 (103)

There were significant differences between the three groups for both t-tau and p-tau. Pairwise comparisons of the age-adjusted medians found significant differences for both t-tau (higher in AD group; median difference 357 pg/ml) and p-tau (higher in AD group; median difference 40.9 pg/ml) between the CAA and AD groups. There was no significant difference between the CAA and CS groups for either t-tau (median difference 53.1 pg/ml) or p-tau (median difference 2.2 pg/ml). There were significant differences between the AD and CS groups for t-tau (higher in AD group; median difference 410 pg/ml) and p-tau (higher in AD group; median difference 43.1 pg/ml).

There was a significant difference in neurogranin between the three groups, but no significant differences were identified in pairwise *post-hoc* comparisons of the adjusted medians. There were no significant differences between the three groups for NFL or sTREM2.

### Aβ ratios

Results for comparison of Aβ ratios are provided in the Supplementary Material.

### Associations with amyloid-PET positivity

We then performed *post-hoc* analyses comparing the CSF profiles of CAA patients with amyloid-PET positive and negative scans. Half of the patients in the CAA group (*n* = 5; 50%) were PET positive by visual read. There were no differences in age, sex or MMSE score between the PET positive and negative groups. When comparing CSF biomarkers (Table 3; Supplementary Figure 3), PET positive CAA patients had lower CSF levels of Aβ_42_ than PET negative CAA patients (median 92.5 versus 134 pg/ml, *p* = 0.047), and higher CSF t-tau (median 440 versus 247 pg/ml, *p* = 0.016), p-tau (median 72.1 versus 45.8 ng/ml, *p* = 0.009), NFL (median 8380 versus 2390 pg/ml, *p* = 0.016), and neurogranin (median 491 versus 349 pg/ml, *p* = 0.016).

**Table 3:** Comparison of PET positive and negative patients with CAA. *p*-values were obtained using one-way ANOVA (age), chi squared (sex) tests, or Wilcoxon-Mann-Whitney tests (remainder)

	PET positive (*n* = 5)	PET negative (*n* = 5)	*p*
Age, y, mean (SD)	69.4 (3.1)	67.8 (2.9)	0.43
Sex, female, *n* (%)	1 (20.0)	1 (20.0)	1.00
MMSE, median (IQR)	29 (29 to 29)	29 (28 to 30)	0.83
Aβ_38_, pg/ml, median (IQR)	2450 (1350 to 2500)	1480 (1440 to 1500)	0.46
Aβ_40_, pg/ml, median (IQR)	4140 (2800 to 4370)	3130 (3040 to 3170)	0.60
Aβ_42_, pg/ml, median (IQR)	92.5 (89.4 to 105)	134 (131 to 140)	0.047
sAβPP*α*, pg/ml, median (IQR)	81.0 (67.9 to 96.2)	98.5 (69.4 to 100)	0.60
sAβPPβ, pg/ml, median (IQR)	82.8 (79.0 to 104)	88.7 (66.3 to 94.1)	0.75
t-tau, pg/ml, median (IQR)	440 (431 to 458)	247 (222 to 258)	0.016
p-tau, pg/ml, median (IQR)	72.1 (70.8 to 87.8)	45.8 (42.1 to 49.1)	0.009
NFL, pg/ml, median (IQR)	8380 (7260 to 10400)	2390 (2230 to 2440)	0.016
sTREM2, pg/ml, median (IQR)	8500 (7300 to 9780)	6660 (5420 to 6780)	0.12
Neurogranin, pg/ml, median (IQR)	491 (489 to 604)	349 (334 to 373)	0.016

Given these results, we performed a further comparison, in which amyloid-PET negative CAA patients were compared with AD and CS groups; these results are provided in the Supplementary Material. Patients with amyloid-PET negative CAA had lower Aβ_38_, Aβ_40_, and Aβ_42_ than both AD and CS groups, with no statistically significant differences between the AD and CS groups for these markers. There were no significant differences in sAβPP*α* and sAβPPβ between the three groups; the results for t-tau, p-tau, NFL, and sTREM2 were similar to those identified in the original analyses. In contrast to the original analysis, neurogranin in amyloid-PET negative CAA was significantly lower than the AD group.

## DISCUSSION

In this exploratory, hypothesis-generating study, we found that patients with CAA had a distinctive CSF profile compared with CS participants and patients with AD. In unadjusted analyses, patients with CAA showed lower levels of all amyloid components measured (Aβ_38_, Aβ_40_, Aβ_42_, sAβPP*α*, and sAβPPβ) and higher NFL, but did not show differences in CSF t-tau, p-tau, sTREM2, or neurogranin profile. Patients with AD had higher t-tau, p-tau and neurogranin than both control participants and patients with CAA. In age-adjusted analyses, differences for the CAA group remained for Aβ_38_, Aβ_40_, Aβ_42_, and sAβPPβ. Finally, we performed exploratory *post-hoc* comparisons within the CAA group, comparing those with amyloid-PET positive and negative scans, and found that those who were PET positive showed differences in Aβ_42_, t-tau, p-tau, NFL, and neurogranin, in an AD-like profile.

Our results for Aβ_40_, Aβ_42_, t-tau, and p-tau in CAA are in keeping with previously reported data [13–19]; however, we extend this earlier work further by demonstrating that Aβ_40_ and Aβ_42_ are not the only amyloid species to be reduced in CAA. The processing pathway from AβPP to pathological Aβ is well described [20–22]. However, CAA differs from AD in that parenchymal Aβ plaques are predominantly composed of Aβ_42_, whereas the vascular Aβ deposits in CAA are a mixture of Aβ_40_ and Aβ_42_, with the former being more common [16, 23]. The reduced levels of CSF Aβ_40_ and Aβ_42_ previously described in CAA are thought to be secondary to “selective trapping” of both these species in the vasculature, in contrast with AD, where only Aβ_42_ is found (“trapped”) in the parenchyma [16]. Aβ peptides of other lengths, including Aβ_38_, have also been shown to be deposited in the leptomeningeal vasculature [24]. Our finding of reductions in CSF Aβ_38_ and sAβPPβ (and sAβPP*α*, in our unadjusted analyses) are novel, and support the protein elimination failure hypothesis for CAA [25], which proposes that CAA results due to failed Aβ clearance via intramural peri-arterial drainage pathways [26]. Our results might suggest that a range of AβPP and Aβ elements are trapped within the cerebral vasculature, potentially the result of a generalized (rather than selective) protein clearance failure. An alternative interpretation is that the reduction in all AβPP and Aβ species we measured reflects decreased AβPP expression, processing and release, rather than altered clearance of the proteins.

Our results also provide new information on non-amyloid biomarkers in CAA. We found significant elevations in t-tau, p-tau, and neurogranin in patients with AD, but not in patients with CAA compared with healthy controls. This is in contrast with other studies which have found that t-tau and p-tau levels in CAA are higher than controls but lower than patients with AD [13, 16, 18, 27], and may reflect our small sample size. Pathological aggregation of tau protein is important in AD [28, 29]; tau aggregation is thought to result in synaptic dysfunction and subsequent neuronal loss, and in AD, it is tau (rather than Aβ) pathology that most closely correlates with cognition [29]. The intermediate tau levels described in CAA might thus be reflective of coexistent AD pathology. Cognitive impairment is a recognized feature of CAA [30] and while there is evidence that CAA is associated with atrophy (presumably secondary to neuronal loss [31]), cognitive impairment in these patients might be secondary to other mechanisms, such as network disruption [32] or impaired blood flow responses [33]. Our CSF findings suggest that synaptic dysfunction is a less prominent feature of CAA compared with AD, and that these markers might be useful for distinguishing these two Aβ pathologies in patients with cognitive impairment.

We also provide new data on other markers that have not been tested in CAA. NFL has shown great promise as a biomarker in a large number of neurological conditions [34], including sporadic cerebral small vessel disease [35–37], although age-adjusted analyses were only performed in one study [36]. We did not find a difference between the AD, CAA, and CS groups in age-adjusted analyses for CSF NFL, but in unadjusted analyses, the CAA group had higher NFL than the control participant group, and in the *post-hoc* analyses, patients with CAA who were amyloid-PET positive had particularly high levels, raising the possibility that this is a useful marker for co-existing AD and CAA pathology. As seems to be the case for many other conditions, including cerebral small vessel disease [37], NFL in CAA is likely to find utility as a marker of the intensity of neurodegeneration and so a useful measure of prognosis rather than diagnosis, particularly as it can be measured in the serum; further work is needed to investigate this. We did not find any differences in sTREM2 between the AD, CAA, or control groups; this is in contrast with previous studies, in which sTREM2 was found to be elevated in the CSF of AD patients [38–42]. Again, our inability to identify a statistically significant difference might result from our relatively small sample size.

Our exploratory *post-hoc* analyses comparing amyloid-PET positive and negative patients with CAA raise new questions about the interactions between AD and CAA pathologies. Although the group sizes are small (with only five participants per group), these findings confirm that not all patients with neuroimaging evidence of CAA are PET positive (in keeping with previous reports; a recent meta-analysis found that amyloid-PET sensitivity in CAA ranged from 60% to 91%) [43], and suggest that there is a sub-group with an AD-like profile—lower CSF Aβ_42_, higher CSF t-tau, phospho-tau, NFL, and neurogranin—who have evidence of additional fibrillary amyloid deposition. As described above, the marked increase in NFL in PET positive CAA patients might suggest that NFL is particularly sensitive for dual AD and CAA pathology. Neurogranin was able to discriminate between PET negative CAA, AD, and CS groups, with elevations only evident in the AD group, providing further evidence that this might be a very specific marker for fibrillary AD pathology. Together, these data support the hypothesis that amyloid-PET positivity in CAA is a measure of co-existent parenchymal Aβ rather than a measure of vascular amyloid and raises the possibility that the degree or extent of co-existent AD pathology in CAA can be measured using these CSF markers. An alternative explanation is that these markers, together with amyloid-PET positivity, are indicative of a higher vascular amyloid burden that results in neurodegeneration via non-AD mechanisms. Further work in larger cohorts is needed, both to replicate these findings and explore these hypotheses further.

Our work has a number of strengths. We were able to evaluate a large number of markers and to reduce false-positive error we have used two separate statistical methods, one rank-based (Kruskal-Wallis) and the other an age-adjusted analysis based upon comparison of medians. However, there are some limitations. As mentioned earlier, this was a small exploratory study which may not have been powered to detect differences for all the biomarkers considered. Given our small sample size, we acknowledge the possibility of false-positive results due to multiple comparisons as a limitation of our work. While larger studies have considered some of the CSF markers we describe (in particular Aβ_40_, Aβ_42_, t-tau, and p-tau), we provide new data in CAA for a number of other markers, which we hope will inform future research with regard to effect sizes and sample size calculations. We acknowledge that there can be discrepancies between clinical and pathological diagnoses of CAA and AD, as well as significant overlap in these pathologies. While we attempted to avoid this on the basis of clinical and radiological findings, we did not have autopsy data, which would be the gold standard. However, we would argue that most clinicians who review patients with AD or CAA make their diagnosis on the basis of clinical and radiological (rather than autopsy) findings, and therefore our results remain of relevance. We specifically selected non-demented CAA patients with “early” or mild disease; these patients may not have such marked biomarker perturbations as those with more severe disease. The AD patients selected from the Specialist Cognitive Disorders Service may not be fully representative of AD patients more generally; this is a highly specialist tertiary service, which often sees younger patients or those with atypical presentations. We used CSF criteria (t-tau and Aβ_42_) in order to identify patients with AD and half of the control group, and so these markers are not fully independent in our analyses. Many of our control participants presented to neurology services (either the TIA clinic, or the Specialist Cognitive Disorders Service), and although we only included individuals without clinical, radiological, or CSF evidence of significant neurological disease, we acknowledge that they cannot be described as true “healthy” controls; this is a further limitation of our work. We selected control participants with minimal radiological evidence of brain pathology (WMH, atrophy), and this may not be truly representative of unselected age-matched individuals without AD or CAA. We determined PET positivity on qualitative (i.e., based on visual reads) rather than quantitative grounds, and the group sizes are particularly small in this *post-hoc* analysis, which precludes from age-adjustment. However, despite these limitations, we provide important new data on these body fluid markers in CAA, and in particular provide data on effect sizes that will be critical for determining sample sizes for larger future studies.

### Conclusions

Our findings suggest that patients with CAA appear to have a CSF profile distinct from both patients with AD and age-matched control participants, characterized by a global reduction in secreted AβPP and Aβ species, but normal neuronal protein levels (tau, neurogranin) compared with AD patients. Replication of these findings in larger cohorts with longitudinal cohort measurements is required to confirm these markers as effective biomarkers for CAA, and their value for monitoring disease progression in clinical trials.

## Supplementary Material

Supplementary Material

## References

[1] Knudsen KA , Rosand J , Karluk D , Greenberg SM (2001) Clinical diagnosis of cerebral amyloid angiopathy: Validation of the Boston criteria. Neurology 56, 537–539. 11222803 10.1212/wnl.56.4.537

[2] Linn J , Halpin A , Demaerel P , Ruhland J , Giese AD , Dichgans M , van Buchem MA , Bruckmann H , Greenberg SM (2010) Prevalence of superficial siderosis in patients with cerebral amyloid angiopathy. Neurology 74, 1346–1350. 20421578 10.1212/WNL.0b013e3181dad605 PMC2875936

[3] Greenberg SM , Al-Shahi Salman R , Biessels GJ , van Buchem M , Cordonnier C , Lee JM , Montaner J , Schneider JA , Smith EE , Vernooij M , Werring DJ (2014) Outcome markers for clinical trials in cerebral amyloid angiopathy. Lancet Neurol 13, 419–428. 24581702 10.1016/S1474-4422(14)70003-1 PMC4085787

[4] Banerjee G , Werring DJ (2019) Feasibility of clinical trial recruitment for cerebral amyloid angiopathy: A specialist single centre experience. J Neurol Sci 409, 116580. 31775058 10.1016/j.jns.2019.116580

[5] Weston PS , Paterson RW , Modat M , Burgos N , Cardoso MJ , Magdalinou N , Lehmann M , Dickson JC , Barnes A , Bomanji JB , Kayani I , Cash DM , Ourselin S , Toombs J , Lunn MP , Mummery CJ , Warren JD , Rossor MN , Fox NC , Zetterberg H , Schott JM (2015) Using florbetapir positron emission tomography to explore cerebrospinal fluid cut points and gray zones in small sample sizes. Alzheimers Dement (Amst) 1, 440–446. 26835507 10.1016/j.dadm.2015.10.001 PMC4691234

[6] Scheltens P , Leys D , Barkhof F , Huglo D , Weinstein HC , Vermersch P , Kuiper M , Steinling M , Wolters EC , Valk J (1992) Atrophy of medial temporal lobes on MRI in “probable” Alzheimer’s disease and normal ageing: Diagnostic value and neuropsychological correlates. J Neurol Neurosurg Psychiatry 55, 967–972. 1431963 10.1136/jnnp.55.10.967 PMC1015202

[7] Pasquier F , Leys D , Weerts JG , Mounier-Vehier F , Barkhof F , Scheltens P (1996) Inter- and intraobserver reproducibility of cerebral atrophy assessment on MRI scans with hemispheric infarcts. Eur Neurol 36, 268–272. 8864706 10.1159/000117270

[8] Fazekas F , Kleinert R , Offenbacher H , Schmidt R , Kleinert G , Payer F , Radner H , Lechner H (1993) Pathologic correlates of incidental MRI white matter signal hyperintensities. Neurology 43, 1683–1689. 8414012 10.1212/wnl.43.9.1683

[9] Elahi FM , Miller BL (2017) A clinicopathological approach to the diagnosis of dementia. Nat Rev Neurol 13, 457–476. 28708131 10.1038/nrneurol.2017.96 PMC5771416

[10] Blennow K , Hampel H , Weiner M , Zetterberg H (2010) Cerebrospinal fluid and plasma biomarkers in Alzheimer disease. Nat Rev Neurol 6, 131–144. 20157306 10.1038/nrneurol.2010.4

[11] Kleinberger G , Yamanishi Y , Suarez-Calvet M , Czirr E , Lohmann E , Cuyvers E , Struyfs H , Pettkus N , Wenninger-Weinzierl A , Mazaheri F , Tahirovic S , Lleo A , Alcolea D , Fortea J , Willem M , Lammich S , Molinuevo JL , Sanchez-Valle R , Antonell A , Ramirez A , Heneka MT , Sleegers K , van der Zee J , Martin JJ , Engelborghs S , Demirtas-Tatlidede A , Zetterberg H , Van Broeckhoven C , Gurvit H , Wyss-Coray T , Hardy J , Colonna M , Haass C (2014) TREM2 mutations implicated in neurodegeneration impair cell surface transport and phagocytosis. Sci Transl Med 6, 243ra286. 10.1126/scitranslmed.3009093 24990881

[12] Lane CA , Parker TD , Cash DM , Macpherson K , Donnachie E , Murray-Smith H , Barnes A , Barker S , Beasley DG , Bras J , Brown D , Burgos N , Byford M , Jorge Cardoso M , Carvalho A , Collins J , De Vita E , Dickson JC , Epie N , Espak M , Henley SMD , Hoskote C , Hutel M , Klimova J , Malone IB , Markiewicz P , Melbourne A , Modat M , Schrag A , Shah S , Sharma N , Sudre CH , Thomas DL , Wong A , Zhang H , Hardy J , Zetterberg H , Ourselin S , Crutch SJ , Kuh D , Richards M , Fox NC , Schott JM (2017) Study protocol: Insight 46 - a neuroscience sub-study of the MRC National Survey of Health and Development. BMC Neurol 17, 75. 28420323 10.1186/s12883-017-0846-x PMC5395844

[13] Renard D , Castelnovo G , Wacongne A , Le Floch A , Thouvenot E , Mas J , Gabelle A , Labauge P , Lehmann S (2012) Interest of CSF biomarker analysis in possible cerebral amyloid angiopathy cases defined by the modified Boston criteria. J Neurol 259, 2429–2433. 22576334 10.1007/s00415-012-6520-8

[14] Tamura R , Tomita H , Mizutani K , Miwa T (2014) The importance of amyloid beta protein in cerebrospinal fluid when you recognize convexal subarachnoid hemorrhage. Eur Neurol 71, 283–287. 24577197 10.1159/000357426

[15] Garcia Estevez DA , Garcia-Dorrego RM , Nieto-Baltar B , Marey-Garrido M , Hierro-Torner T (2017) Spontaneous convexity subarachnoid haemorrhage: Clinical series of 3 patients with associated cerebral amyloid angiopathy. Neurologia 32, 213–218. 26778730 10.1016/j.nrl.2015.11.004

[16] Verbeek MM , Kremer BP , Rikkert MO , Van Domburg PH , Skehan ME , Greenberg SM (2009) Cerebrospinal fluid amyloid beta(40) is decreased in cerebral amyloid angiopathy. Ann Neurol 66, 245–249. 19743453 10.1002/ana.21694 PMC3697750

[17] Martinez-Lizana E , Carmona-Iragui M , Alcolea D , Gomez-Choco M , Vilaplana E , Sanchez-Saudinos MB , Clarimon J , Hernandez-Guillamon M , Munuera J , Gelpi E , Gomez-Anson B , de Juan-Delago M , Delgado-Mederos R , Montaner J , Ois A , Amaro S , Blesa R , Marti-Fabregas J , Lleo A , Fortea J (2015) Cerebral amyloid angiopathy-related atraumatic convexal subarachnoid hemorrhage: An ARIA before the tsunami. J Cereb Blood Flow Metab 35, 710–717. 25735919 10.1038/jcbfm.2015.25 PMC4420868

[18] Charidimou A , Friedrich JO , Greenberg SM , Viswanathan A (2018) Core cerebrospinal fluid biomarker profile in cerebral amyloid angiopathy: A meta-analysis. Neurology 90, e754–e762. 29386280 10.1212/WNL.0000000000005030 PMC5837868

[19] van Etten ES , Verbeek MM , van der Grond J , Zielman R , van Rooden S , van Zwet EW , van Opstal AM , Haan J , Greenberg SM , van Buchem MA , Wermer MJ , Terwindt GM (2017) beta-Amyloid in CSF: Biomarker for preclinical cerebral amyloid angiopathy. Neurology 88, 169–176. 27903811 10.1212/WNL.0000000000003486 PMC5224714

[20] Kummer MP , Heneka MT (2014) Truncated and modified amyloid-beta species. Alzheimers Res Ther 6, 28. 25031638 10.1186/alzrt258 PMC4055046

[21] Selkoe DJ (1991) The molecular pathology of Alzheimer’s disease. Neuron 6, 487–498. 1673054 10.1016/0896-6273(91)90052-2

[22] Hardy JA , Higgins GA (1992) Alzheimer’s disease: The amyloid cascade hypothesis. Science 256, 184–185. 1566067 10.1126/science.1566067

[23] Iwatsubo T , Odaka A , Suzuki N , Mizusawa H , Nukina N , Ihara Y (1994) Visualization of A beta 42(43) and A beta 40 in senile plaques with end-specific A beta monoclonals: Evidence that an initially deposited species is A beta 42(43). Neuron 13, 45–53. 8043280 10.1016/0896-6273(94)90458-8

[24] Kakuda N , Miyasaka T , Iwasaki N , Nirasawa T , Wada-Kakuda S , Takahashi-Fujigasaki J , Murayama S , Ihara Y , Ikegawa M (2017) Distinct deposition of amyloid-beta species in brains with Alzheimer’s disease pathology visualized with MALDI imaging mass spectrometry. Acta Neuropathol Commun 5, 73. 29037261 10.1186/s40478-017-0477-x PMC5641992

[25] Carare RO , Hawkes CA , Jeffrey M , Kalaria RN , Weller RO (2013) Review: Cerebral amyloid angiopathy, prion angiopathy, CADASIL and the spectrum of protein elimination failure angiopathies (PEFA) in neurodegenerative disease with a focus on therapy. Neuropathol Appl Neurobiol 39, 593–611. 23489283 10.1111/nan.12042

[26] Albargothy NJ , Johnston DA , MacGregor-Sharp M , Weller RO , Verma A , Hawkes CA , Carare RO (2018) Convective influx/glymphatic system: Tracers injected into the CSF enter and leave the brain along separate periarterial basement membrane pathways. Acta Neuropathol 136, 139–152. 29754206 10.1007/s00401-018-1862-7 PMC6015107

[27] Renard D , Gabelle A , Hirtz C , Demattei C , Thouvenot E , Lehmann S (2016) Cerebrospinal fluid Alzheimer’s disease biomarkers in isolated supratentorial cortical superficial siderosis. J Alzheimers Dis 54, 1291–1295. 27567848 10.3233/JAD-160400

[28] Ballatore C , Lee VM , Trojanowski JQ (2007) Tau-mediated neurodegeneration in Alzheimer’s disease and related disorders. Nat Rev Neurosci 8, 663–672. 17684513 10.1038/nrn2194

[29] Ittner A , Ittner LM (2018) Dendritic tau in Alzheimer’s disease. Neuron 99, 13–27. 30001506 10.1016/j.neuron.2018.06.003

[30] Banerjee G , Carare R , Cordonnier C , Greenberg SM , Schneider JA , Smith EE , Buchem MV , Grond JV , Verbeek MM , Werring DJ (2017) The increasing impact of cerebral amyloid angiopathy: Essential new insights for clinical practice. J Neurol Neurosurg Psychiatry 88, 982–994. 28844070 10.1136/jnnp-2016-314697 PMC5740546

[31] Fotiadis P , van Rooden S , van der Grond J , Schultz A , Martinez-Ramirez S , Auriel E , Reijmer Y , van Opstal AM , Ayres A , Schwab KM , Alzheimer’s Disease Neuroimaging I, Hedden T , Rosand J , Viswanathan A , Wermer M , Terwindt G , Sperling RA , Polimeni JR , Johnson KA , van Buchem MA , Greenberg SM , Gurol ME (2016) Cortical atrophy in patients with cerebral amyloid angiopathy: A case-control study. Lancet Neurol 15, 811–819. 27180034 10.1016/S1474-4422(16)30030-8 PMC5248657

[32] Reijmer YD , Fotiadis P , Martinez-Ramirez S , Salat DH , Schultz A , Shoamanesh A , Ayres AM , Vashkevich A , Rosas D , Schwab K , Leemans A , Biessels GJ , Rosand J , Johnson KA , Viswanathan A , Gurol ME , Greenberg SM (2015) Structural network alterations and neurological dysfunction in cerebral amyloid angiopathy. Brain 138, 179–188. 25367025 10.1093/brain/awu316 PMC4285191

[33] Peca S , McCreary CR , Donaldson E , Kumarpillai G , Shobha N , Sanchez K , Charlton A , Steinback CD , Beaudin AE , Fluck D , Pillay N , Fick GH , Poulin MJ , Frayne R , Goodyear BG , Smith EE (2013) Neurovascular decoupling is associated with severity of cerebral amyloid angiopathy. Neurology 81, 1659–1665. 24097810 10.1212/01.wnl.0000435291.49598.54 PMC3812103

[34] Zetterberg H (2016) Neurofilament light: A dynamic cross-disease fluid biomarker for neurodegeneration. Neuron 91, 1–3. 27387643 10.1016/j.neuron.2016.06.030

[35] Jonsson M , Zetterberg H , van Straaten E , Lind K , Syversen S , Edman A , Blennow K , Rosengren L , Pantoni L , Inzitari D , Wallin A (2010) Cerebrospinal fluid biomarkers of white matter lesions - cross-sectional results from the LADIS study. Eur J Neurol 17, 377–382. 19845747 10.1111/j.1468-1331.2009.02808.x

[36] Duering M , Konieczny MJ , Tiedt S , Baykara E , Tuladhar AM , Leijsen EV , Lyrer P , Engelter ST , Gesierich B , Achmuller M , Barro C , Adam R , Ewers M , Dichgans M , Kuhle J , de Leeuw FE , Peters N (2018) Serum neurofilament light chain levels are related to small vessel disease burden. J Stroke 20, 228–238. 29886723 10.5853/jos.2017.02565 PMC6007291

[37] Gattringer T , Pinter D , Enzinger C , Seifert-Held T , Kneihsl M , Fandler S , Pichler A , Barro C , Grobke S , Voortman M , Pirpamer L , Hofer E , Ropele S , Schmidt R , Kuhle J , Fazekas F , Khalil M (2017) Serum neurofilament light is sensitive to active cerebral small vessel disease. Neurology 89, 2108–2114. 29046363 10.1212/WNL.0000000000004645 PMC5711505

[38] Gispert JD , Suarez-Calvet M , Monte GC , Tucholka A , Falcon C , Rojas S , Rami L , Sanchez-Valle R , Llado A , Kleinberger G , Haass C , Molinuevo JL (2016) Cerebrospinal fluid sTREM2 levels are associated with gray matter volume increases and reduced diffusivity in early Alzheimer’s disease. Alzheimers Dement 12, 1259–1272. 27423963 10.1016/j.jalz.2016.06.005

[39] Heslegrave A , Heywood W , Paterson R , Magdalinou N , Svensson J , Johansson P , Ohrfelt A , Blennow K , Hardy J , Schott J , Mills K , Zetterberg H (2016) Increased cerebrospinal fluid soluble TREM2 concentration in Alzheimer’s disease. Mol Neurodegener 11, 3. 26754172 10.1186/s13024-016-0071-x PMC4709982

[40] Piccio L , Deming Y , Del-Aguila JL , Ghezzi L , Holtzman DM , Fagan AM , Fenoglio C , Galimberti D , Borroni B , Cruchaga C (2016) Cerebrospinal fluid soluble TREM2 is higher in Alzheimer disease and associated with mutation status. Acta Neuropathol 131, 925–933. 26754641 10.1007/s00401-016-1533-5 PMC4867123

[41] Suarez-Calvet M , Kleinberger G , Araque Caballero MA , Brendel M , Rominger A , Alcolea D , Fortea J , Lleo A , Blesa R , Gispert JD , Sanchez-Valle R , Antonell A , Rami L , Molinuevo JL , Brosseron F , Traschutz A , Heneka MT , Struyfs H , Engelborghs S , Sleegers K , Van Broeckhoven C , Zetterberg H , Nellgard B , Blennow K , Crispin A , Ewers M , Haass C (2016) sTREM2 cerebrospinal fluid levels are a potential biomarker for microglia activity in early-stage Alzheimer’s disease and associate with neuronal injury markers. EMBO Mol Med 8, 466–476. 26941262 10.15252/emmm.201506123 PMC5120370

[42] Suarez-Calvet M , Araque Caballero MA , Kleinberger G , Bateman RJ , Fagan AM , Morris JC , Levin J , Danek A , Ewers M , Haass C (2016) Early changes in CSF sTREM2 in dominantly inherited Alzheimer’s disease occur after amyloid deposition and neuronal injury. Sci Transl Med 8, 369ra178. 10.1126/scitranslmed.aag1767 PMC5385711 27974666

[43] Charidimou A , Farid K , Baron JC (2017) Amyloid-PET in sporadic cerebral amyloid angiopathy: A diagnostic accuracy meta-analysis. Neurology 89, 1490–1498. 28855406 10.1212/WNL.0000000000004539

