# Supplementary material for: Cerebrospinal Fluid Biomarkers in Cerebral Amyloid Angiopathy
Source: J Alzheimers Dis. 2020 Apr 21;74(4):1189–201. doi: 10.3233/JAD-191254 (PMC7242825; doi:10.3233/JAD-191254)

Supplementary Material

Cerebrospinal Fluid Biomarkers in Cerebral Amyloid Angiopathy

SUPPLEMENTARY RESULTS

Aβ ratios

$A\beta_{42}$:$A\beta_{40}$ ratio

There was a significant difference between the three groups (Supplementary Table 1; Supplementary Figure 2A; p=0.0001). In post-hoc comparisons, patients with CAA had significantly lower CSF $A\beta_{42}$:$A\beta_{40}$ ratio than the CS group (corrected p=0.00003), but not the AD group (corrected p=0.06). Patients with AD had a lower $A\beta_{42}$:$A\beta_{40}$ ratio than the CS group (corrected p=0.0036). In age-adjusted quantile regression, there was a significant difference between the three groups (p=0.0003). Pairwise comparison of the medians found significant differences between CAA and CS groups, and the AD and CS groups. The difference between the CAA and AD groups did not reach statistical significance.

$A\beta_{40}$: $A\beta_{38}$ ratio

There was a significant difference between the three groups (Supplementary Table 1; Supplementary Figure 2B; p=0.0001). In post-hoc comparisons, patients with CAA had significantly lower CSF $A\beta_{40}$:$A\beta_{38}$ ratio than the AD group (corrected p=0.0039), but not the CS group (corrected p=0.22). There was no significant difference between the AD and CS groups (corrected p=0.28). In age-adjusted quantile regression, there was no significant difference between the three groups (p=0.77).

$A\beta_{42}$: $A\beta_{38}$ ratio

There was a significant difference between the three groups (Supplementary Table 1; Supplementary Figure 1C; $p=0.0003$). In post-hoc comparisons, patients with CAA had significantly lower CSF $A\beta_{42}$: $A\beta_{38}$ ratio than both the CS (corrected $p=0.00003$) and AD (corrected $p=0.0273$) groups. Patients with AD had a lower CSF $A\beta_{42}$: $A\beta_{38}$ ratio than the CS group (corrected $p=0.0345$). In age-adjusted quantile regression, there was as significant difference between the three groups ($p=0.0013$). Pairwise comparison of the medians found significant differences between CAA and CS groups and the AD and CS groups. The difference between the CAA and AD groups did not reach statistical significance.

Exploratory comparisons with amyloid-negative CAA group

Having performed post-hoc analyses comparing the CSF profiles of CAA patients with amyloid-PET positive and negative scans, we performed a further comparison, in which amyloid-PET negative CAA patients were compared with AD and CS groups. Comparisons between amyloid-PET negative CAA patients, the AD and CS groups were made using the Kruskal-Wallis and then Dunn's test for post-hoc comparisons (for group comparisons with $p<0.05$).

We found similar results to those identified in the full analysis (where comparisons were made with all CAA patients); patients with amyloid-PET negative CAA still had lower $A\beta_{38}$, $A\beta_{40}$, and $A\beta_{42}$ than both AD ($A\beta_{38}$ corrected $p=0.0021$, $A\beta_{40}$ corrected $p=0.0015$, $A\beta_{42}$ corrected $p=0.0138$) and CS ($A\beta_{38}$ corrected $p=0.0036$, $A\beta_{40}$ corrected $p=0.0036$, $A\beta_{42}$ corrected $p=0.0003$) groups, with no statistically significant differences between the AD and CS groups for

all three markers. There were no significant differences in sAβPPα and sAβPPβ between the three groups, and the results for t-tau, p-tau, NFL, and sTREM2 were similar to those identified in the original analyses. However, in contrast to the original analysis, neurogranin in amyloid-PET negative CAA was significantly lower than the AD group (corrected $p=0.0036$). Comparison of Aβ ratios found similar results (i.e., statistically significant reductions in $A\beta_{42}$: $A\beta_{40}$ and $A\beta_{42}$: $A\beta_{38}$ ratios for the CAA and AD groups compared with the CS group) to the original analysis.

Supplementary Table 1. Univariable and age-adjusted quantile regression for Aβ ratios. Univariable p-values are from Kruskal-Wallis tests.

Ratio	Group	Univariable comparison		Age-adjusted quantile regression		
		Median (IQR)	p	β (SE)	Predicted median, (95% CI)	p
$A\beta_{42}$: $A\beta_{40}$ ratio	CAA	0.032 (0.028 to 0.042)	0.0001	*Reference group*	0.034 (0.019 to 0.048)	0.0003
	AD	0.049 (0.040 to 0.055)		0.015 (0.01)	0.049 (0.040 to 0.058)	
	CS	0.076 (0.068 to 0.110)		0.043 (0.01)	0.077 (0.064 to 0.090)	
$A\beta_{40}$: $A\beta_{38}$ ratio	CAA	2.069 (1.777 to 2.209)	0.0104	*Reference group*	2.201 (1.994 to 2.408)	0.7714
	AD	2.375 (2.174 to 2.477)		0.095 (0.13)	2.295 (2.164 to 2.427)	
	CS	2.312 (2.020 to 2.393)		0.066 (0.15)	2.267 (2.083 to 2.451)	
$A\beta_{42}$: $A\beta_{38}$ ratio	CAA	0.068 (0.050 to 0.095)	0.0003	*Reference group*	0.086 (0.046 to 0.126)	0.0013
	AD	0.114 (0.091 to 0.138)		0.025 (0.03)	0.112 (0.086 to 0.137)	
	CS	0.181 (0.130 to 0.268)		0.101 (0.03)	0.187 (0.152 to 0.223)	

Supplementary Figure 1. Scatter plots of each biomarker by age.

A) Aβ38

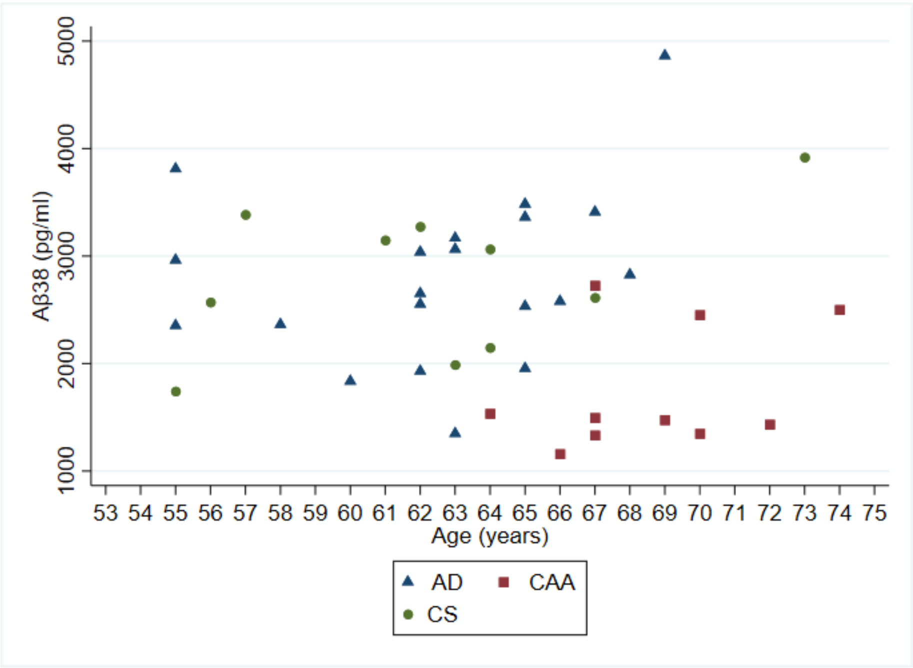

B) Aβ40

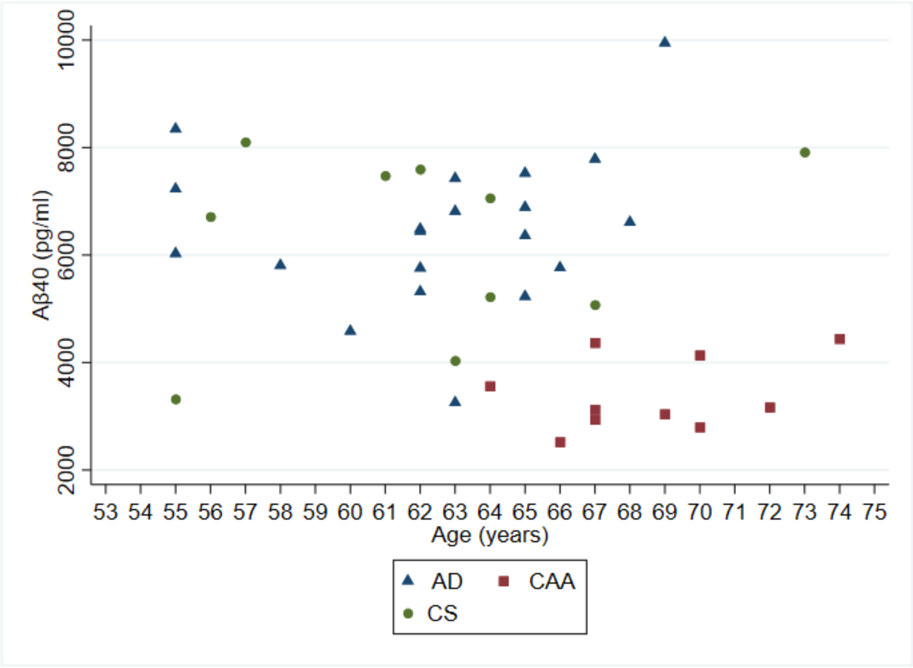

C) $A\beta_{42}$

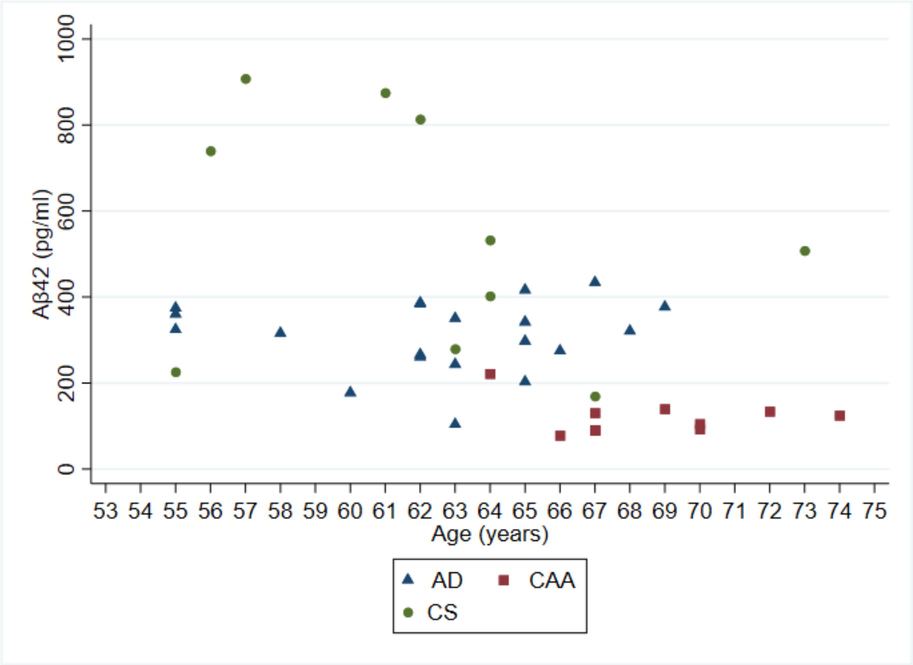

D) sAβPPα

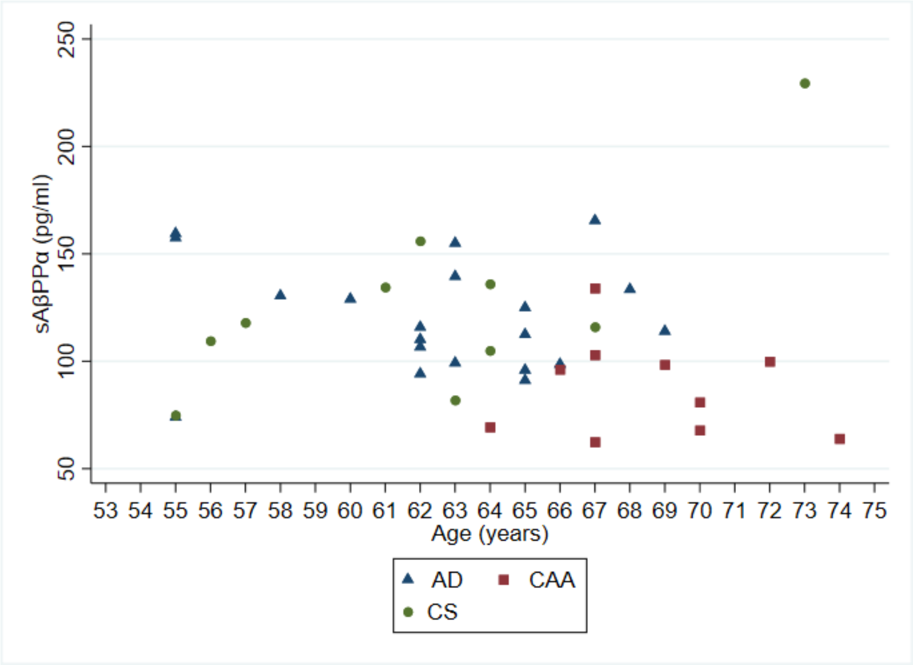

E) sAβPPβ

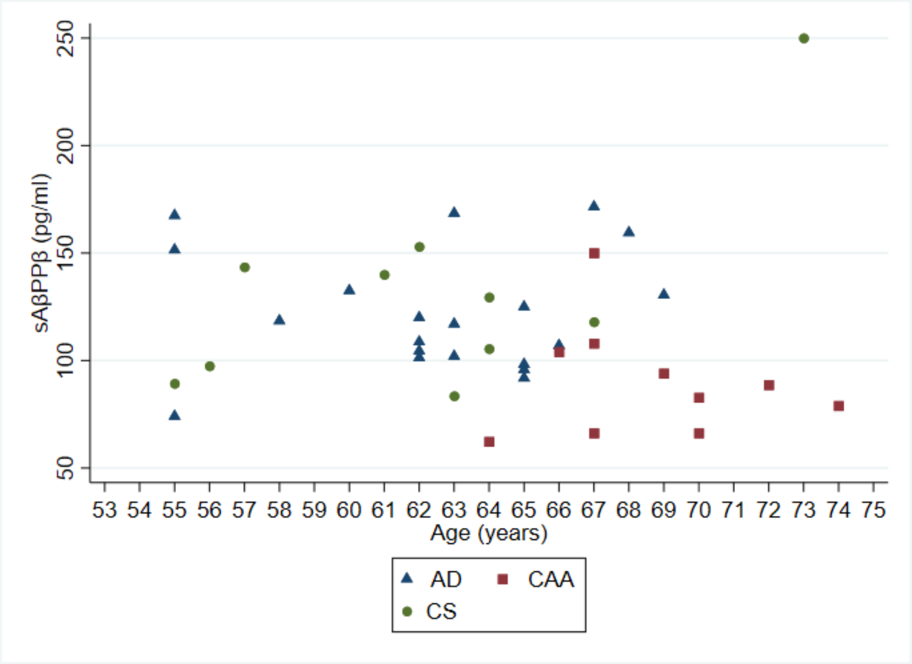

F) Total tau

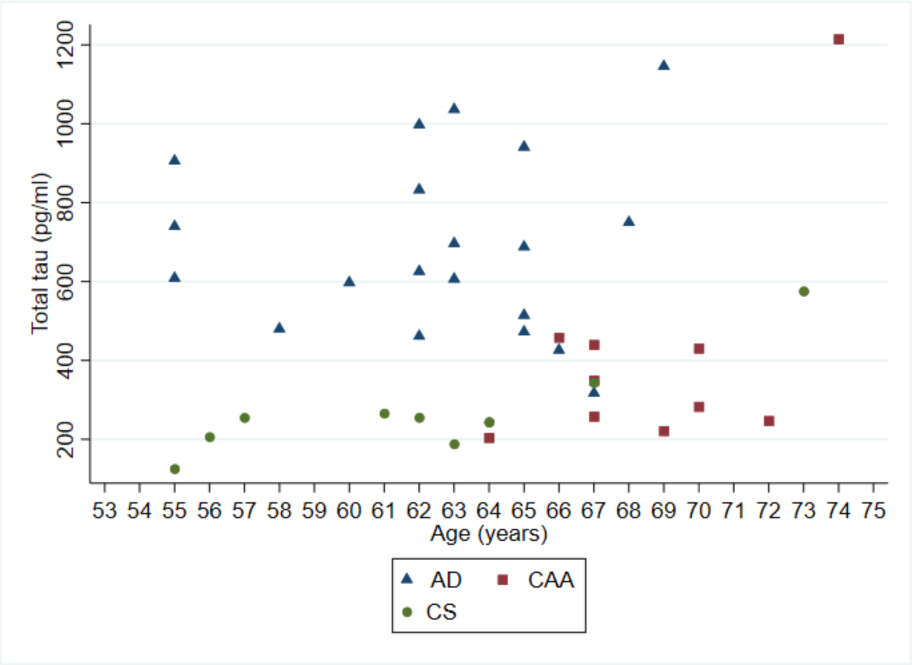

G) Phospho-tau

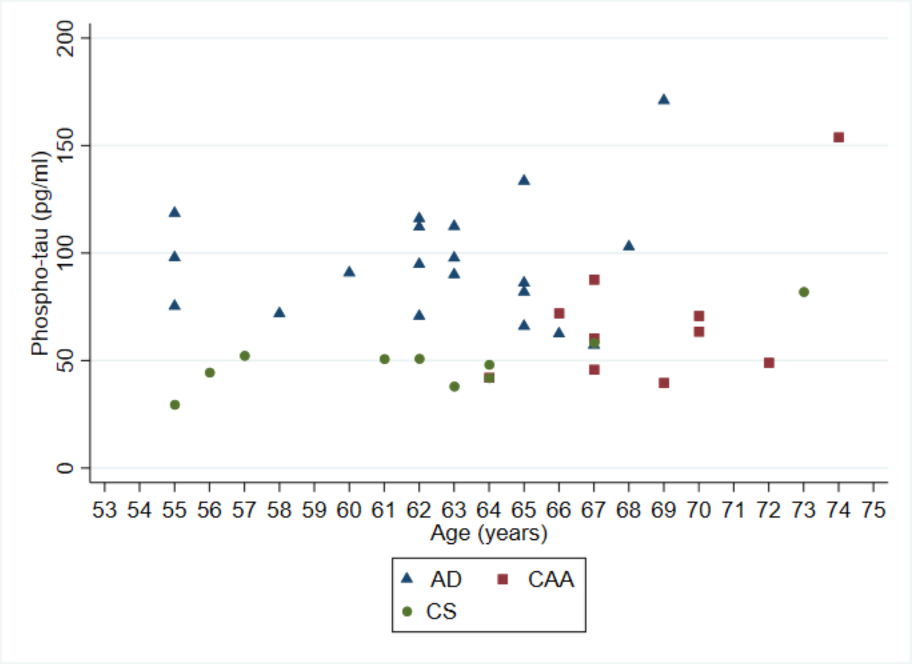

H) Neurofilament light

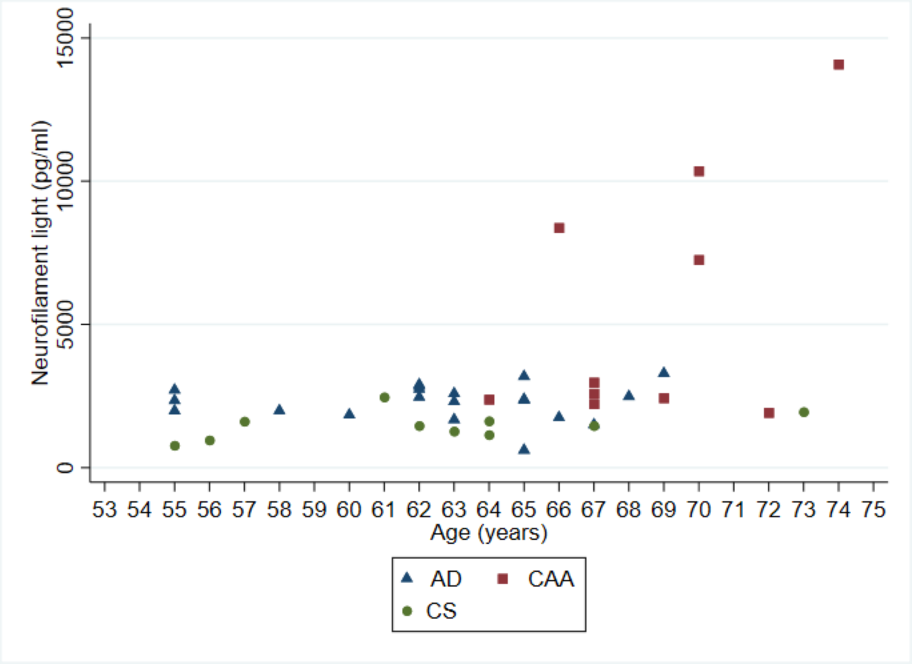

I) sTREM2

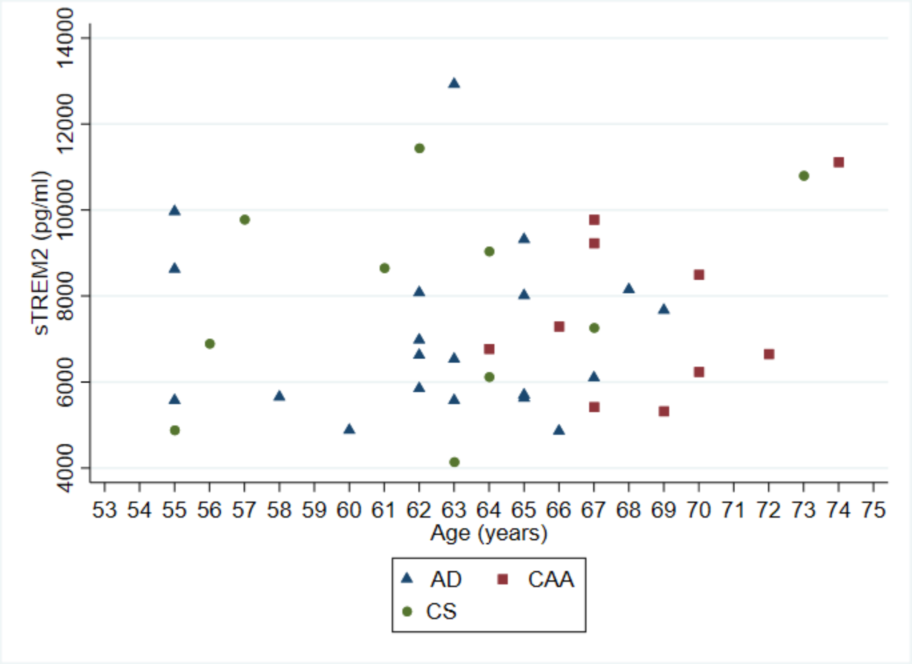

J) Neurogranin

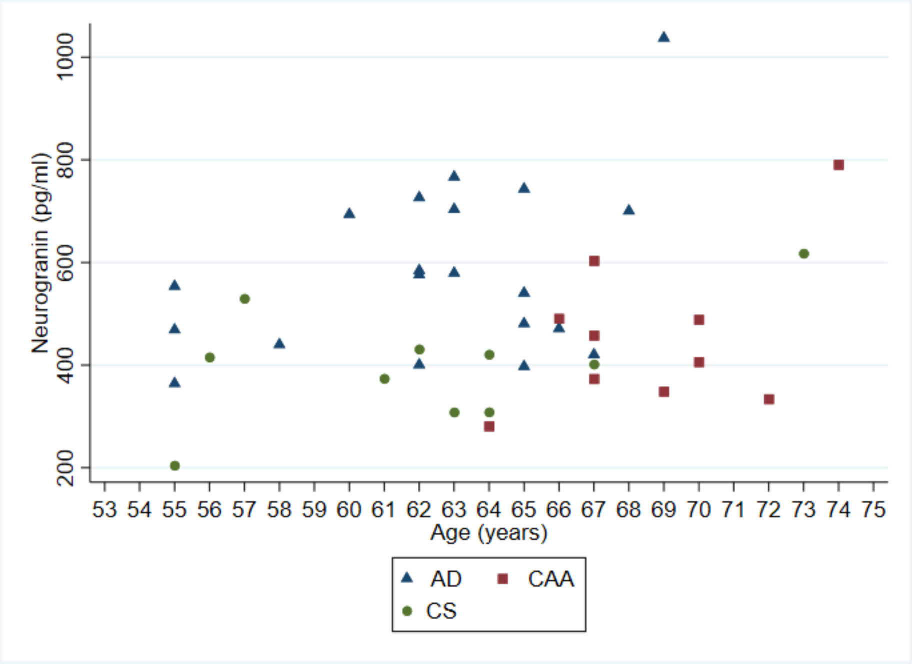

Supplementary Figure 2. Comparison of Aβ ratios in AD, CAA, and control participant (CS) groups. Each diamond indicates an individual data point. p values are derived from post-hoc Dunn's test and have been Bonferroni-corrected. *$p \leq 0.05$; **$p \leq 0.01$; ***$p \leq 0.001$; ****$p \leq 0.0001$

A) $A\beta_{42}$: $A\beta_{40}$ ratio

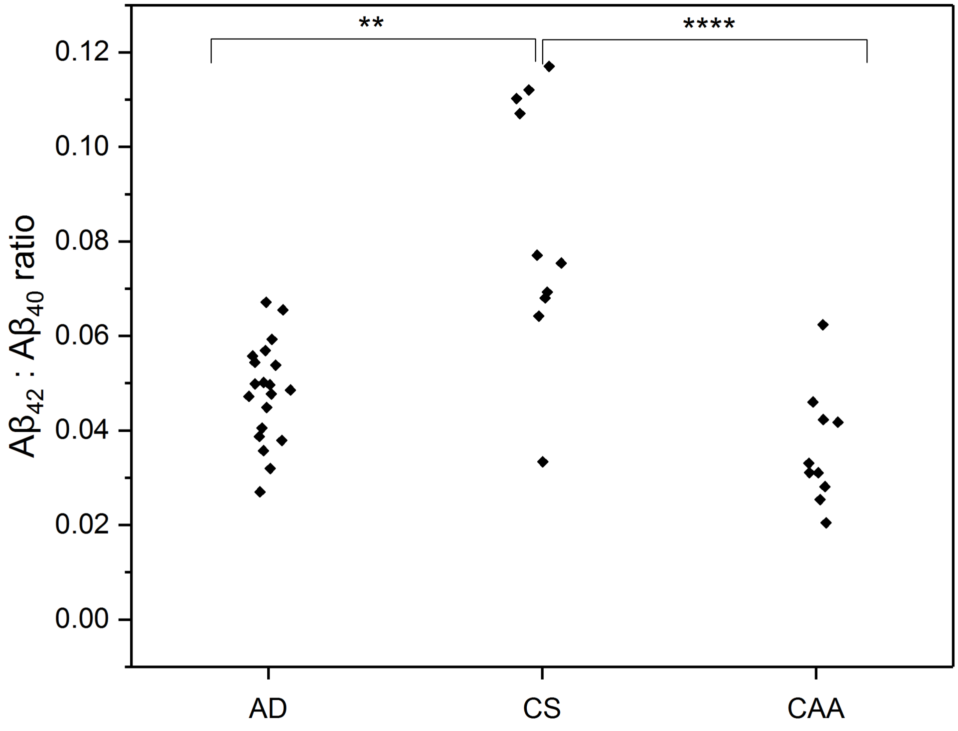

B) Aβ$_{40}$: Aβ$_{38}$ ratio

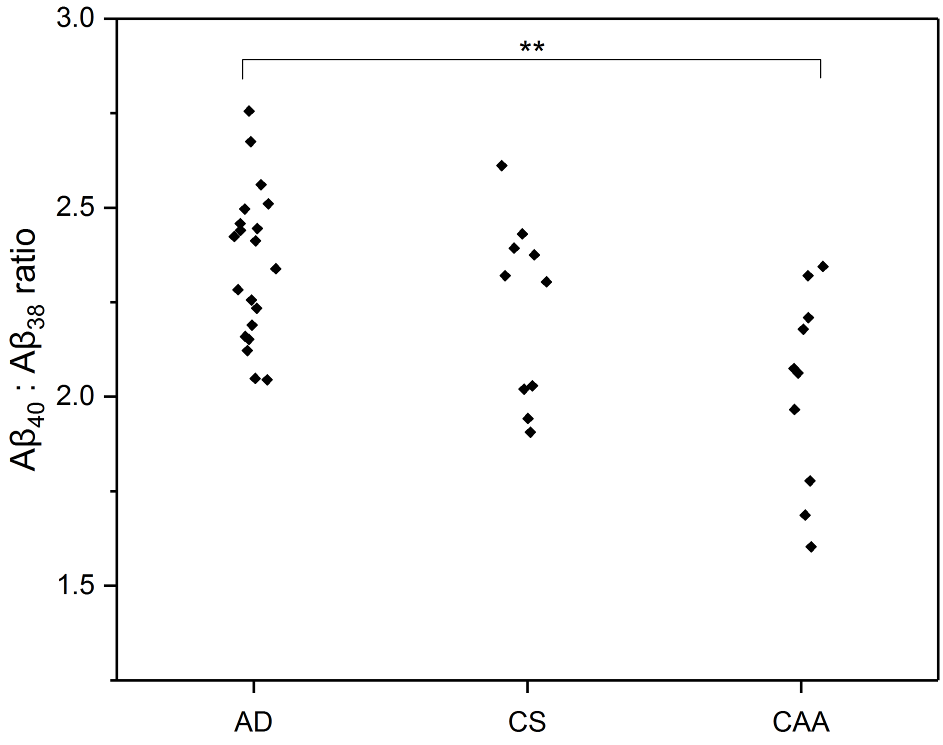

C) $A\beta_{42}$: $A\beta_{38}$ ratio

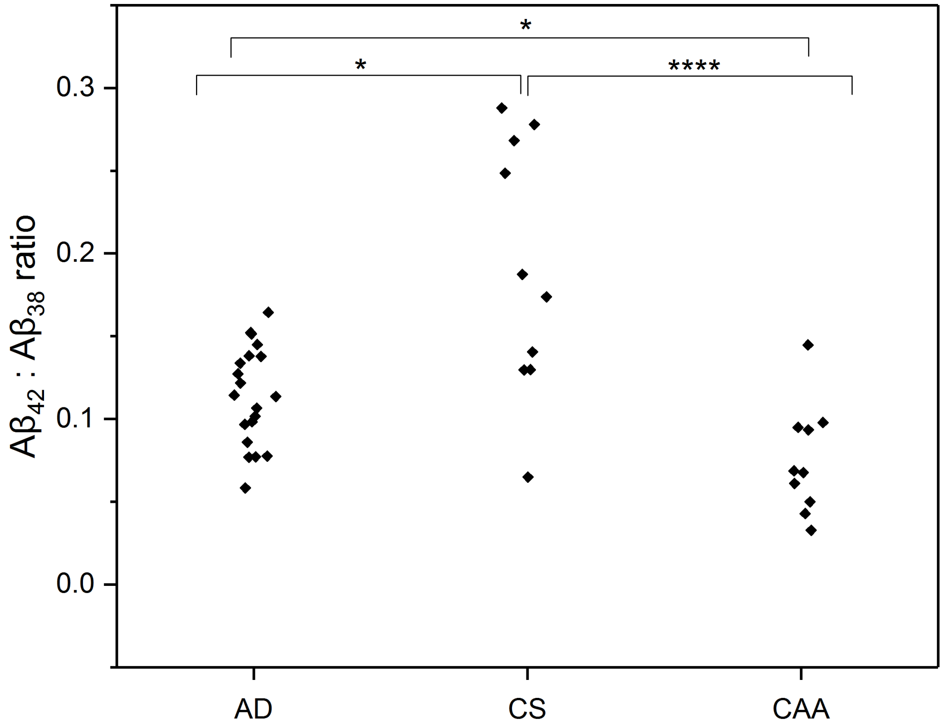

Supplementary Figure 3. Comparison of CSF biomarker profiles in PET positive and negative patients with CAA. Horizontal line indicates median value per group. Each diamond indicates an individual data point.

A) Aβ_{38}

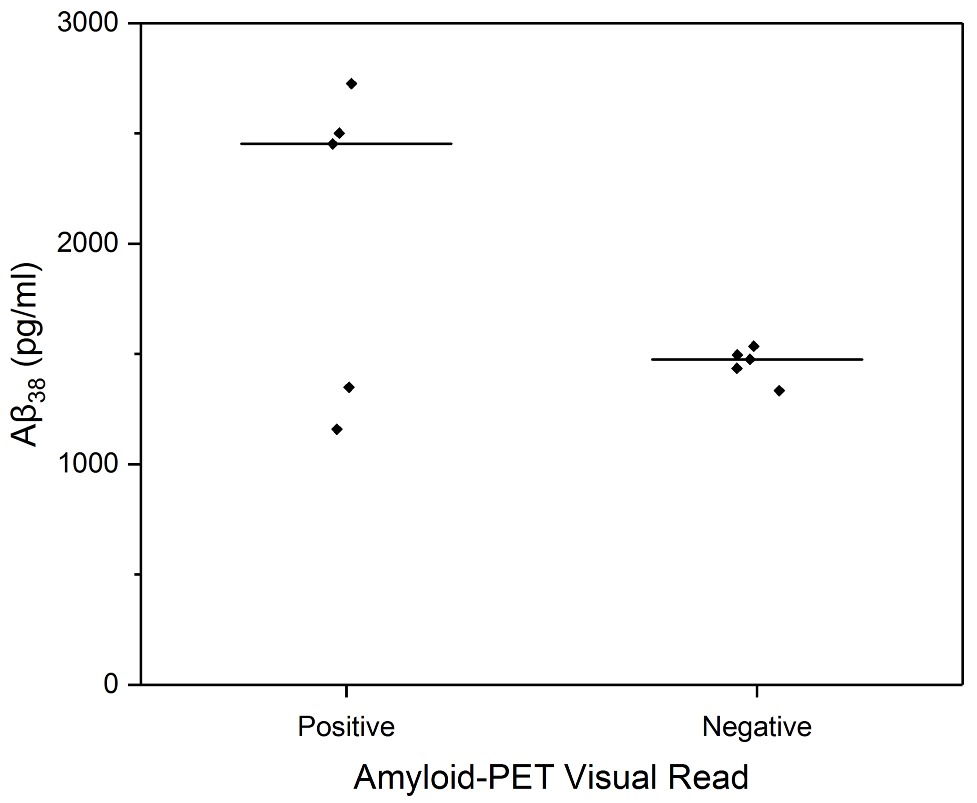

B) Aβ$_{40}$

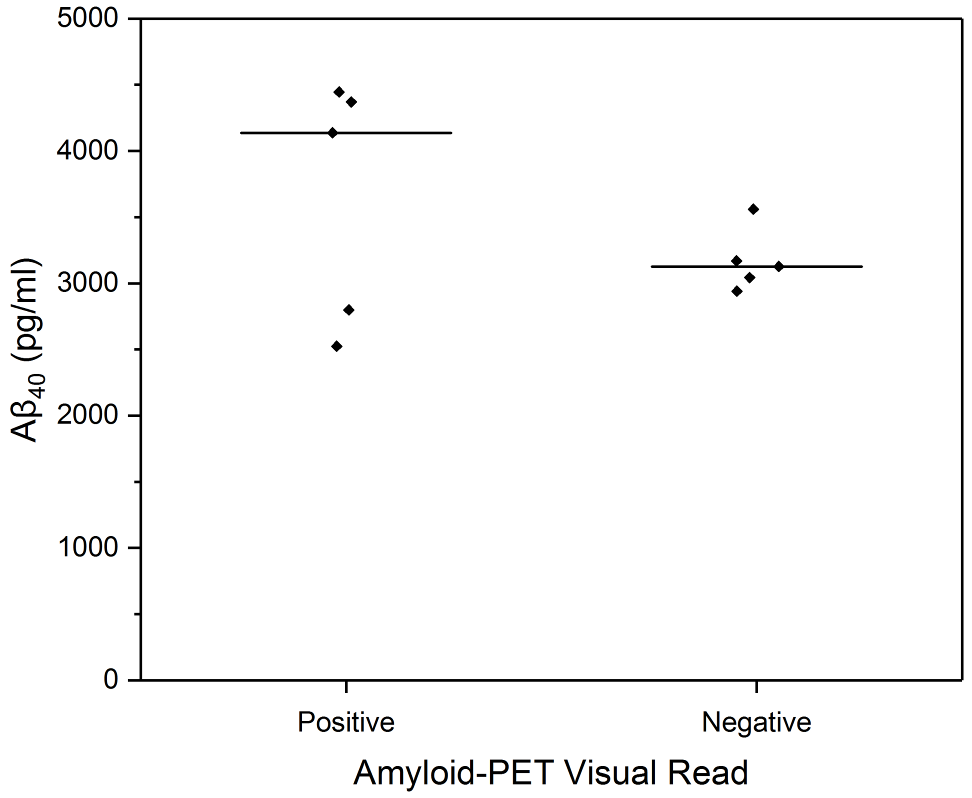
5000
4000
3000
2000
1000
0
Aβ40 (pg/ml)
Positive
Negative
Amyloid-PET Visual Read

C) Aβ_{42}

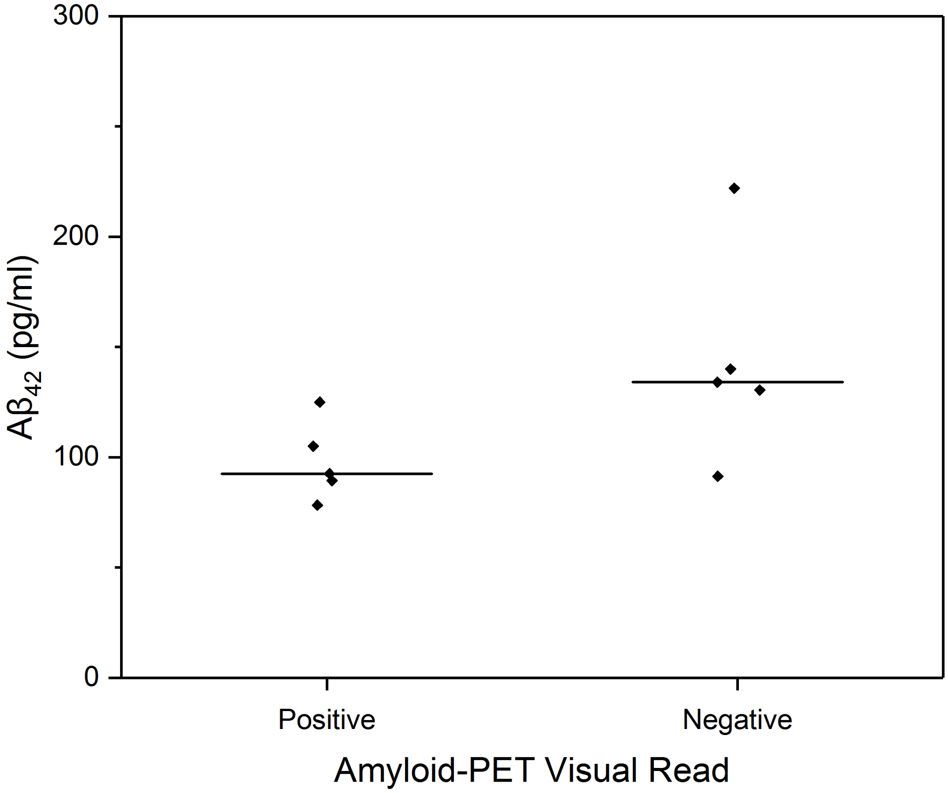
300
200
100
0
Aβ42 (pg/ml)
Positive
Negative
Amyloid-PET Visual Read

D) sAβPPα

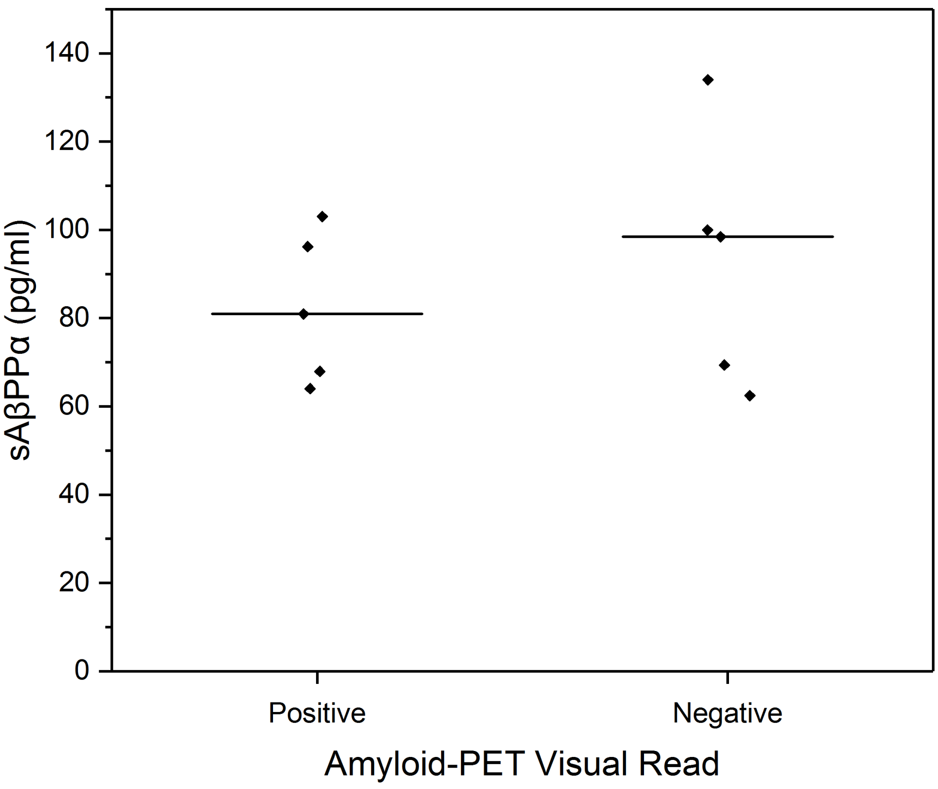

sAβPPα (pg/ml)
0
20
40
60
80
100
120
140
Positive
Negative
Amyloid-PET Visual Read

E) sAβPPβ

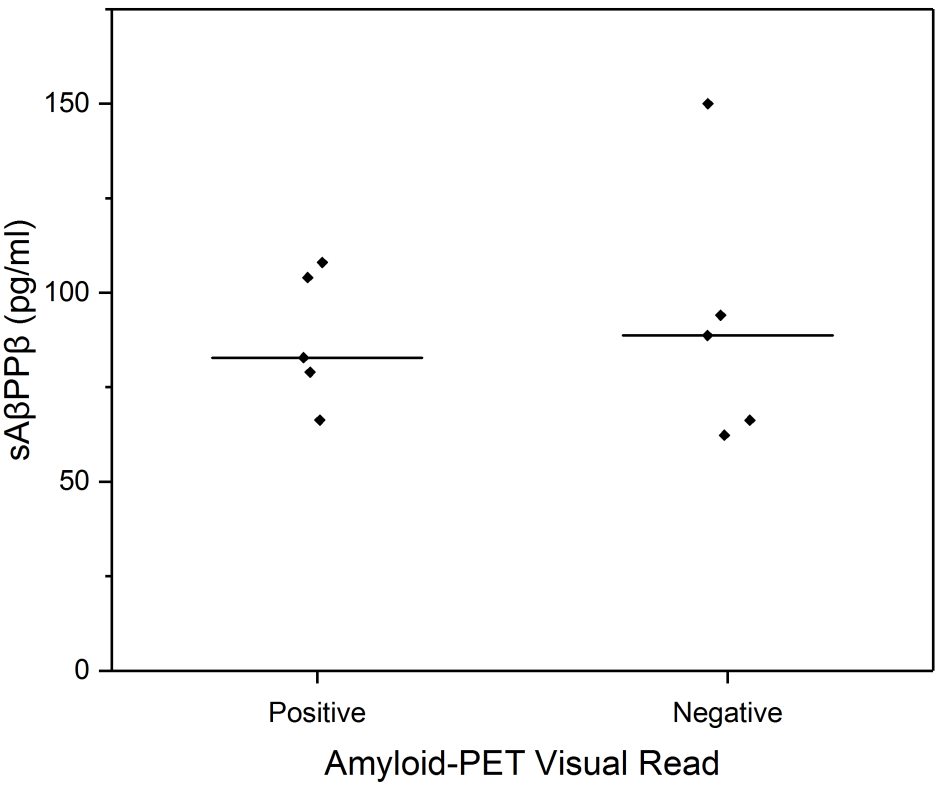

sAβPPβ (pg/ml)
150
100
50
0
Positive
Negative
Amyloid-PET Visual Read

F) t-tau

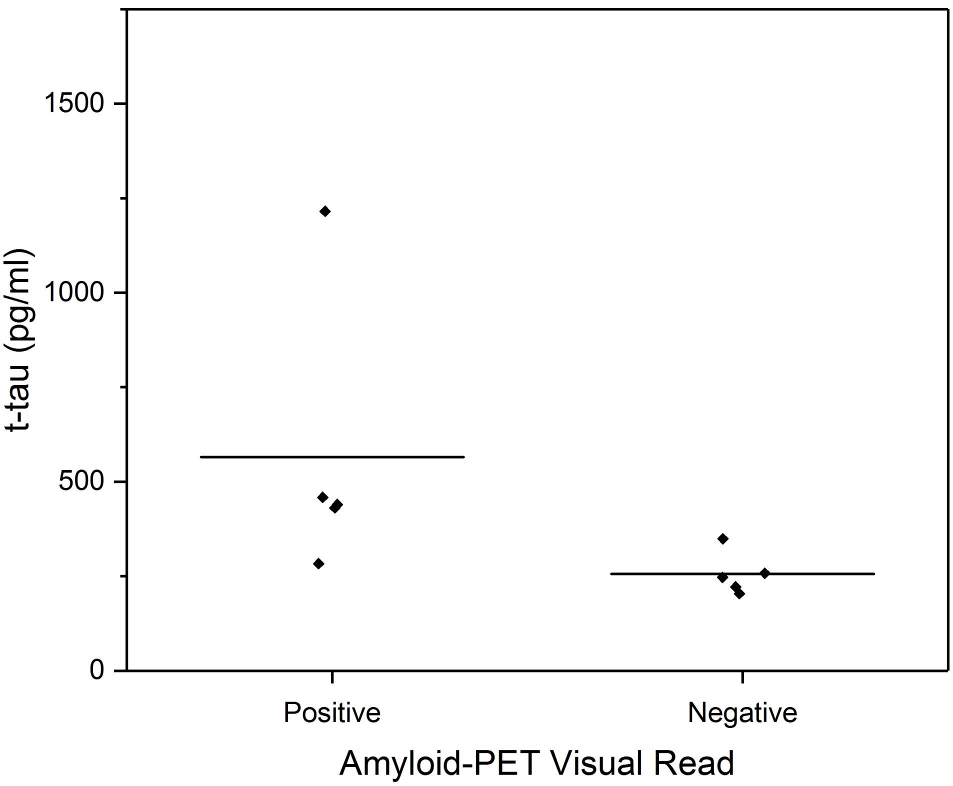

1500
1000
500
0
t-tau (pg/ml)
Positive
Negative
Amyloid-PET Visual Read

G) p-tau

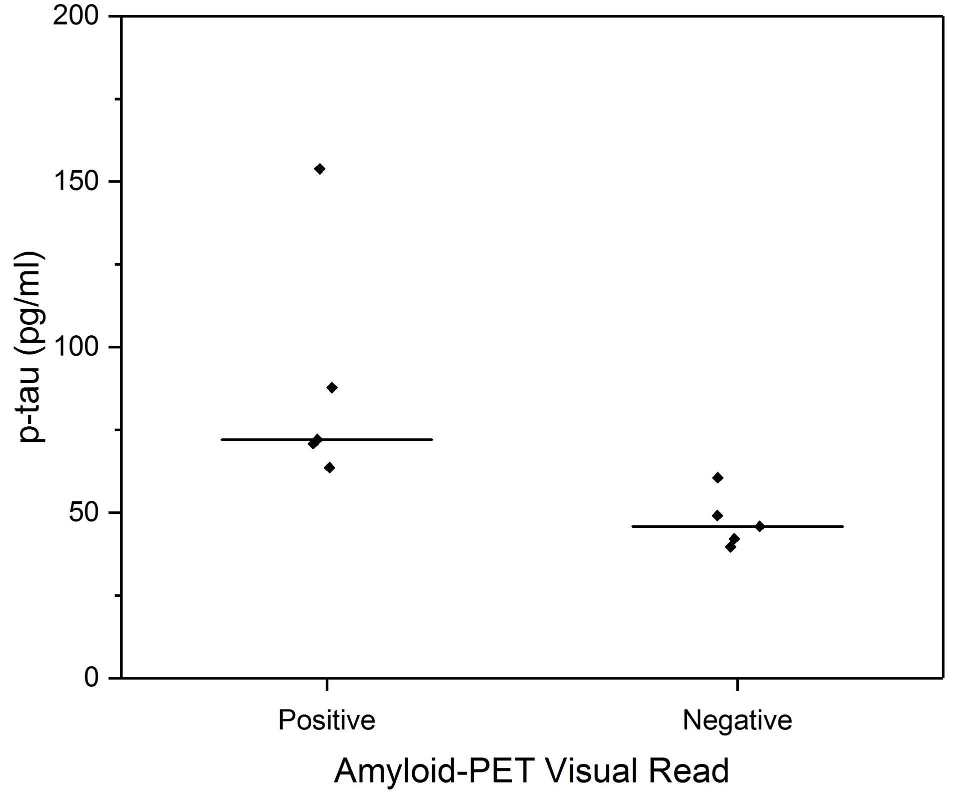
200
150
100
50
0
p-tau (pg/ml)
Positive
Negative
Amyloid-PET Visual Read

H) NFL

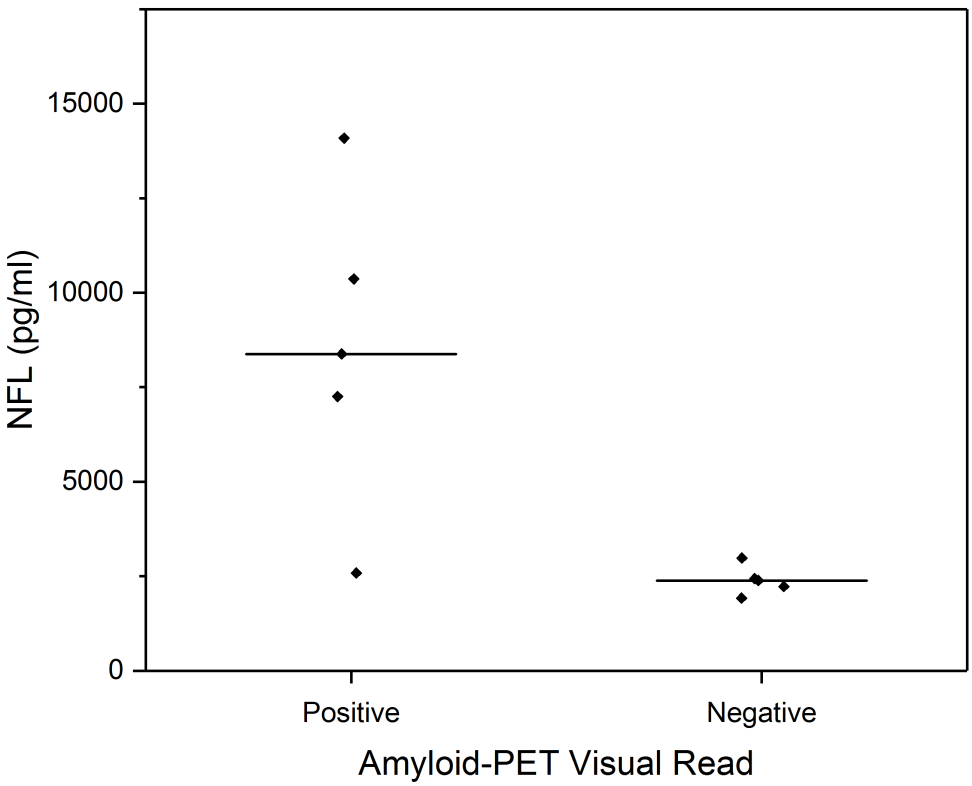

NFL (pg/ml)
15000
10000
5000
0
Positive
Negative
Amyloid-PET Visual Read

I) sTREM2

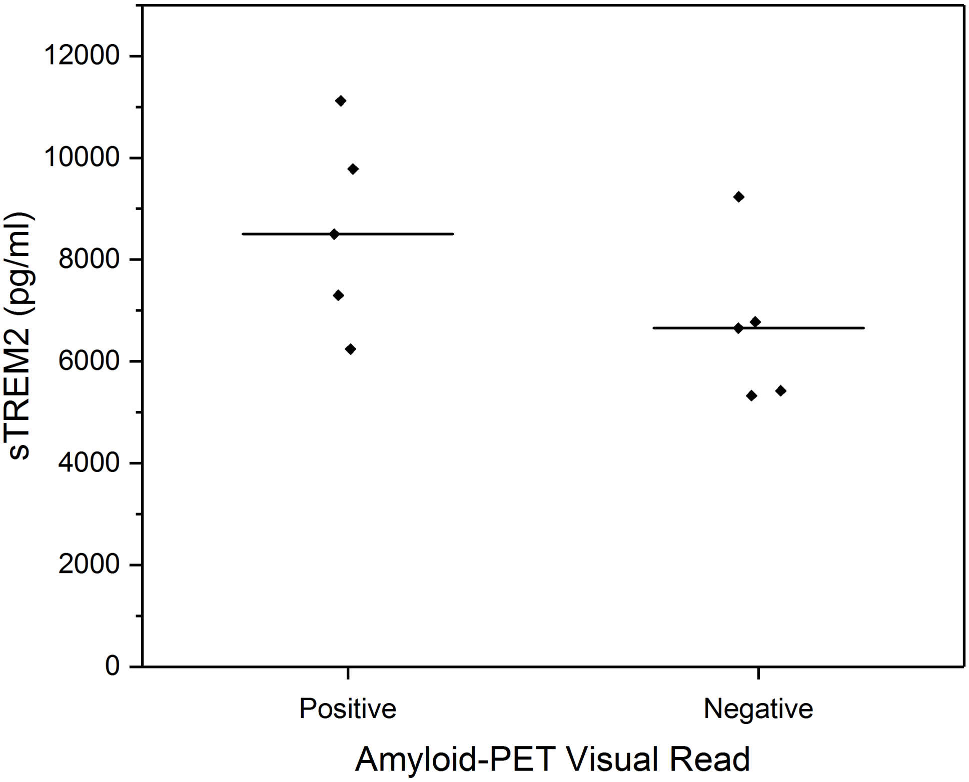

12000
10000
8000
6000
4000
2000
0
sTREM2 (pg/ml)
Positive
Negative
Amyloid-PET Visual Read

J) Neurogranin

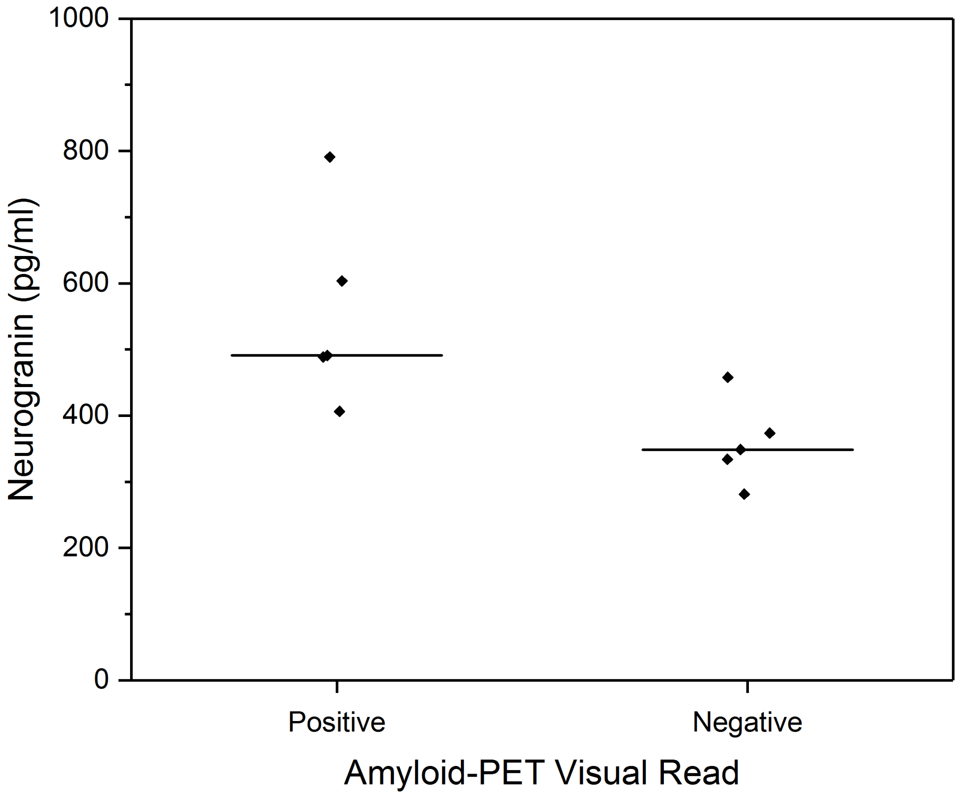

Supplement: Supplementary Material [file jad-74-jad191254-s001.pdf]
